# Glossary of terms for musculoskeletal radiology

**DOI:** 10.1007/s00256-020-03465-1

**Published:** 2020-06-02

**Authors:** William Palmer, Laura Bancroft, Fiona Bonar, Jung-Ah Choi, Anne Cotten, James F. Griffith, Philip Robinson, Christian W.A. Pfirrmann

**Affiliations:** 1 Department of Radiology, Massachusetts General Hospital, Harvard Medical School, Boston, USA; 2 Radiology Associates of Venice and Englewood, Venice, Florida USA; 3 Douglass Hanly Moir Pathology, Notre Dame Medical School, Royal Prince Alfred Hospital, Sydney, Australia; 4 Department of Radiology, Hallym University College of Medicine, Hallym University Dongtan Sacred Heart Hospital, Hwaseong, Gyeonggi-do South Korea; 5 Service de Radiologie at Imagerie Musculosquelettique, Centre de Consultations et d’Imagerie de l’Appareil Locomoteur, Lille, France; 6 Department of Imaging and Interventional Radiology, Prince of Wales Hospital, The Chinese University of Hong Kong, Hong Kong, China; 7 Radiology Department, Leeds Teaching Hospitals, Chapel Allerton Hospital, University of Leeds and NHIR Biomedical Research Unit, Leeds, UK; 8 MRI Medical Radiological Institute, Zurich, Switzerland

**Keywords:** Musculoskeletal radiology, Musculoskeletal disorders, Musculoskeletal anatomy, Glossary

## Abstract

Members of the International Skeletal Society compiled a glossary of terms for musculoskeletal radiology. The authors also represent national radiology or pathology societies in Asia, Australia, Europe, and the USA. We provide brief descriptions of musculoskeletal structures, disease processes, and syndromes and address their imaging features. Given the abundance of musculoskeletal disorders and derangements, we chose to omit most terms relating to neoplasm, spine, intervention, and pediatrics. Consensus agreement was obtained from 19 musculoskeletal radiology societies worldwide.

## Introduction

This glossary comprises terms for musculoskeletal radiology. It focuses on the anatomical structures, disease processes, and syndromes that are fundamental to the musculoskeletal lexicon. A goal of this glossary was the presentation of authoritative terminology and knowledge. Terms were vetted and endorsed globally by leaders in musculoskeletal radiology and pathology. At the inception of this project, we obtained formal support from the presidents of 11 societies of musculoskeletal radiology during the annual meeting of the International Skeletal Society in Berlin. At its completion, we solicited input and obtained consensus agreement from 19 societies of musculoskeletal radiology.

The authors compiled a list of potential terms and selected 101 for inclusion in the glossary. Although some terms were first described decades ago, they have evolved in meaning as new knowledge has arrived. We prioritized terms that might be problematic in meaning for residents, fellows, general radiologists, and even subspecialty musculoskeletal radiologists. Given the abundance of musculoskeletal disorders and derangements, we chose to omit most terms relating to neoplasm, spine, intervention, and pediatrics.

This glossary incorporates orthopedic syndromes and rheumatological diseases that are primarily diagnosed applying a combination of clinical, laboratory, and imaging criteria. Most of the impingement syndromes fall into this category. Imaging findings can support these diagnoses but may be inconclusive in the absence of appropriate symptoms, signs, or histopathology results. In these particular syndromes and diseases, radiologists are challenged by complicated imaging features that are ambiguous and often reported inconsistently in the literature.

We concentrated on written terminology and scientific precision. Our descriptions distill knowledge and promote a musculoskeletal lexicon. Despite our careful review of the published literature, we realize that words are subjective, and meanings are inherently controversial. Individuals may disagree with our nomenclature and explanation. We included standard imaging techniques used in clinical practice and excluded experimental techniques used in research. Given our large number of terms, we eliminated illustrations because many terms would require multiple images for useful depiction. Illustrations are available through reference publications, textbooks, on-line resources, and the ISS website.

We circulated our glossary among the presidents of 19 societies specializing in musculoskeletal radiology to request endorsement and provide opportunities for input worldwide. Some presidents sought backing from executive committee members who contributed valuable feedback and substantially improved our definitions. After reviewing the glossary, the following societies chose to endorse formally its terminology.

Arabian Gulf Society of Skeletal Radiology

Asian Musculoskeletal Society

Australian Musculoskeletal Imaging Group

British Society of Skeletal Radiologists

Chinese Society of Musculoskeletal Radiology

Deutschen Gesellschaft für Muskuloskelettale Radiologie

European Society of Skeletal Radiology

Indonesian Society of Musculoskeletal Radiology

International Skeletal Society

Japanese Society of Musculoskeletal Radiology

Korean Society of Skeletal Radiology

Musculoskeletal Radiology Society of Chinese Medical Doctors Association

Musculoskeletal Society of India

Sociedad Española de Radiología Musculoesquelética

Saudi Society of Skeletal Radiology

Société d’Imagerie Musculo-Squelettique

Society of Skeletal Radiology

South African Musculoskeletal Imaging Group

Swiss Society of Skeletal Radiology

## Glossary

### Acetabular labral tear

#### Pathogenesis

Acetabular labral tear is a defect of the labral surface, intralabral substance, or chondrolabral junction. Certain conditions predispose to labral tearing in young patients, such as femoroacetabular impingement and congenital hip dysplasia. Labral tears are often asymptomatic [1]. Symptomatic tears are usually located anterosuperiorly along the acetabular rim. Degenerative tears are common in older patients with osteoarthritis. The diagnostic gold standard is arthroscopy or open surgery.

#### Imaging

On MR, the major criterion for tear is fluid disrupting the labral contour or chondrolabral junction [2]. Diagnostic confidence is increased in the presence of labral displacement, chondral lesion, capsular stripping, or paralabral cyst. On arthrographic MR and CT, contrast material outlines the labrum and chondrolabral junction, distends the perilabral recess, and fills tears (excluding intrasubstance tears) [2, 3]. In young adults, tears are linear and sharply defined. They can be classified based on length and location using a quadrant or clock-face system. The sublabral sulcus (cleft, recess), a normal developmental variation, partially undercuts the labrum at the chondrolabral junction and can be mistaken for tear [3] (see also “Hip impingement, femoroacetabular (FAI)”).

### Acetabular retroversion

#### Pathogenesis

Acetabular retroversion refers to abnormal posterior angulation of the superolateral acetabular rim. It usually reflects anterosuperior prominence rather than posterosuperior deficiency. Malorientation can be developmental (acetabular dysplasia) or acquired (acetabular ossicle). It is associated with pincer-type femoroacetabular impingement due to focal femoral overcoverage [4, 5]. Surgical treatments include periacetabular osteotomy and acetabular rim reconstruction (osteochondroplasty).

#### Imaging

Radiographic assessment requires precise centering techniques. On anteroposterior radiographs of the pelvis, the crossover and ischial spine signs are most specific for acetabular retroversion and focal anterosuperior overcoverage. The crossover sign is positive when the anterosuperior rim projects lateral to the posterosuperior rim. The ischial spine sign is positive when the ischial spine projects medial to the pelvic brim. The posterior wall sign assesses prominence or deficiency of the posterior wall. Acetabular version can be measured on transverse CT and MR images. CT 3D reconstructions enable surgical planning and virtual simulations of motion, impingement, and treatment outcomes (see also “Version,” “Hip impingement, femoroacetabular (FAI),” “Hip impingement, pincer deformity”).

### Adductor splints

See “Femoral diaphyseal stress injury.”

### Adhesive capsulitis

#### Histopathology

Adhesive capsulitis (arthrofibrosis, frozen shoulder) refers to progressive, painful restriction in passive and active glenohumeral motion. It is often a clinical diagnosis of exclusion. When primary (idiopathic), it predominates in women older than 40 years. It is associated with diabetes and autoimmune disease. When secondary, it usually results from trauma, surgery, or immobilization. In earlier (freezing) stages, capsular inflammation involves the rotator interval [6]. In later (frozen) stages, inflammation is replaced by fibrosis [7].

#### Imaging

Radiographs may be normal. Arthrography shows diminished joint capacity. On oblique sagittal MR images, thickened soft tissue surrounds the coracohumeral ligament and effaces subcoracoid fat in the rotator interval [6–8]. The axially pouch may also be thickened. Active, painful capsulitis is associated with T2W signal increase, contrast enhancement, pericapsular edema, and bursal effusion [9]. Chronic arthrofibrosis shows T2W signal decrease and pericapsular scarring [8]. Image-guided treatments include intra-articular corticosteroid injection and distension arthrography (brisement).

### Ankle, anterior (anteromedial, anterolateral) impingement

#### Pathogenesis

Anterior and anteromedial ankle impingement syndromes are primarily caused by abnormal bony proliferation along the tibiotalar joint line [10]. Mechanical symptoms and pain result from chronic osseous abutment and secondary capsular entrapment. Anterolateral impingement syndrome is primarily caused by capsular thickening, synovial meniscoid lesion, and hypertrophic scarring between the lateral malleolus, tibia, and talus [11]. These soft tissue abnormalities are often associated with repeated high or low lateral ankle sprains [12]. Therefore, patients may present with symptoms of both impingement and instability.

#### Imaging

In anterior and anteromedial impingements, radiographs, CT, and MR show “kissing” bony proliferation involving the tibial plafond and dorsal talus along capsular attachment sites. Marginal osteophytes may occur in similar locations in asymptomatic patients. In anterolateral impingement, MR demonstrates thickened soft tissue distorting the anterolateral gutter [13]. Localized edema and scar-like tissue increase MR specificity, but impingement syndrome remains a clinical diagnosis (see also “Impingement syndrome,” “Ankle, posterior (posteromedial) impingement”).

### Ankle, posterior (posteromedial) impingement

#### Pathogenesis

Posterior ankle impingement is an overuse syndrome associated with repetitive plantar flexion [14]. Cumulative soft tissue compression between the tibia, talus, and calcaneus results in capsulitis, synovitis, and painful hypertrophic scarring. Impingement is exacerbated by an enlarged lateral talar process (Stieda’s process) or os trigonum. In contrast, posteromedial ankle impingement syndrome follows acute traumatic injury to the posterior tibiotalar ligament and adjacent posteromedial capsule [15]. During the healing process, soft tissue thickening may lead to chronic painful entrapment, inflammation, and hypertrophic scarring between the posterior malleolus and talus.

#### Imaging

In posterior impingement, MR shows capsular thickening and localized edema or fluid in the posterior and posterolateral joint recesses. Bone marrow edema (BME)-like signal may involve the posterior calcaneus, lateral talar process, or os trigonum [16]. In posteromedial impingement, MR shows thickened soft tissue distorting the posteromedial gutter [17]. Although localized edema or inflammation can increase MR specificity, impingement syndrome remains a clinical diagnosis (see also “Impingement syndrome,” “Ankle, anterior (anteromedial, anterolateral) impingement”).

### Anterolateral ligament of the knee

#### Anatomy

The anterolateral ligament (ALL) of the knee courses anteriorly from the lateral femoral epicondyle to the lateral tibial rim. It has firm connections to the lateral meniscus. Proximally, it merges with the lateral collateral ligament (LCL) [18]. Distally, it attaches to the tibia between the fibular head and Gerdy’s tubercle. It functions synergistically with the anterior cruciate ligament (ACL), resisting internal rotation and anterior tibial translation. ACL injuries are associated with ALL injuries and tibial rim avulsion (Segond fracture) [19].

#### Imaging

On MR, the ALL is more difficult to identify than the cruciate and collateral ligaments. The ALL can sometimes be followed distally from its femoral attachment near the LCL. Whereas the LCL is always present, the ALL may not be visible as a discrete ligamentous structure due to normal developmental variation [20]. In the setting of ACL rupture, inspect MR images more closely for synergistic ALL injuries such as lateral meniscal tear and Segond fracture [19].

### Athletic pubalgia

See “Groin pain.”

### Atypical femoral fracture

#### Histopathology

Atypical femoral fracture refers to a femoral diaphyseal stress fracture that results from prolonged (> 3 years) therapy using a bisphosphonate or other antiresorptive agent. Bisphosphonates induce osteoclast apoptosis and decrease the risk of fragility fracture in postmenopausal osteoporosis by suppressing osteoclast-mediated bone resorption and curbing the loss of cancellous bone [21]. Other antiresorptive mechanisms include the inhibition of osteoclast recruitment, maturation, and function [21]. In cortical bone, however, decreased osteoclast activity can disrupt microfracture repair in regions of mechanical stress [22, 23]. Accumulated microdamage leads to atypical, tensile-sided stress fracture of the femoral diaphysis. It is “atypical” because osteoporotic fractures rarely involve the lateral femoral cortex. Due to poor healing potential and risk for fracture completion, patients may undergo prophylactic surgical fixation [24].

#### Imaging

On radiographs, findings include focal periosteal reaction and linear transverse lucency at the lateral cortex of the mid-proximal femoral diaphysis. The risk of catastrophic fracture completion increases with medial propagation of the stress fracture [19]. Bilateral femoral radiographs may demonstrate asymptomatic contralateral fracture requiring prophylactic fixation. When radiographs are negative, CT and MR improve fracture detection in symptomatic patients on bisphosphonate therapy [23, 24]. Similar to other tensile-sided stress fractures, healing is delayed [22]. Acute fracture completion shows a distinctive transverse orientation and minimal comminution [23] (see also “Osteoporosis,” “Looser zone”).

### Avulsion fracture

#### Pathogenesis

Avulsion fracture is a focal, traumatic detachment of bone due to tensile forces transmitted through a tendon, ligament, or joint capsule. Common sites include the humeral tuberosities, olecranon process, phalangeal base, iliac spines, ischial tuberosity, tibial plateau, lateral tibial rim, fibular head, and fifth metatarsal base [25]. Whereas impaction fracture may be depressed due to compressive mechanism, avulsion fracture is often distracted due to tensile mechanism.

#### Imaging

On radiographs and CT, the avulsed bone fragment is nondisplaced or displaced from parent bone in the direction of the attached tendon, ligament, or joint capsule. On MR, the Segond fracture and other cortically based avulsion fractures are easily missed due to small size and the absence of cancellous bone [26]. Adjacent soft tissue edema or hemorrhage may obscure the fracture fragment. Avulsion fracture is associated with minimal or absent BME-like signal due to the tensile mechanism and lack of bone contusion [27] (see also “Bone bruise”, “Bone marrow edema,” “Osteochondral defect,” “Subchondral insufficiency fracture”).

### Bisphosphonate-related fracture

See “Atypical femoral fracture.”

### Bone bruise

#### Pathogenesis

Bone bruise is an acute traumatic injury of cancellous bone. Synonyms include bone marrow contusion and trabecular microfracture. Histology shows trabecular disorganization, interstitial hemorrhage, and fat necrosis [28]. It results from osseous impaction and the transmission of excessive load across the subchondral bone plate or cortex. In weight-bearing joints, subchondral bone bruises may result from twisting injuries (e.g., femorotibial pivot shift). In nonweight-bearing joints, subcortical bone bruises may result from dislocation–impaction injuries (e.g., transient glenohumeral or patellofemoral dislocation) [27]. Healing potential and prognosis are excellent in the absence of chondral injury or cortical fracture [29].

#### Imaging

Radiographs are negative for acute cancellous abnormality but may show cortical depression due to dislocation–impaction fracture (e.g., Hill–Sachs fracture) [30]. MR shows BME-like signal without trabecular fracture line [27, 31]. Subchondral bone bruise (microfracture, hemorrhage, and fat necrosis due to acute injury) and stress response (interstitial edema and reparative microcallus due to cumulative overload) may have identical appearances despite histological and mechanistic differences [29]. Differential diagnosis includes subchondral insufficiency fracture and osteochondral lesions (see also “Bone marrow edema,” “Subchondral insufficiency fracture,” “Osteochondral defect,” “Avulsion fracture”).

### Bone island

#### Histopathology

Bone islands (enostoses) are benign, asymptomatic lesions of compact bone located ectopically within cancellous bone. Histologically, they consist of dense, mature lamellar bone with an extensive Haversian system and, therefore, may be categorized as hamartomas. Peripheral spicules merge radially into surrounding trabeculae. Active remodeling is possible, explaining enostoses that slowly increase or decrease in size. In tubular bones, bone islands are common incidental findings that have a predilection for the epiphysis. They often involve the pelvis, proximal femur, and ribs. Osteopoikilosis is a sclerosing bone dysplasia demonstrating multiple bilateral bone islands in a periarticular distribution [32].

#### Imaging

Radiographic and CT findings are usually pathognomonic [33]. Densely mineralized, spiculated foci are sharply demarcated against surrounding cancellous bone. The spicules follow trabecular lines. On MR, all sequences show signal voids similar to cortical bone. Bone islands are typically small (< 1 cm) and incidental. Giant bone islands (> 2 cm) can demonstrate bone scan activity and central heterogeneity suspicious for malignancy. CT attenuation measurements may help to differentiate bone islands from untreated sclerotic metastases [34]. The differential diagnosis of solitary bone island includes osteoid osteoma, osteoblastoma, osteosarcoma, and osteoblastic metastasis. The differential diagnosis of multiple bone islands includes tuberous sclerosis, osteopoikilosis, osteoblastic metastases, and treated metastases.

### Bone marrow edema

#### Histopathology

BME refers to nonspecific MR signal increase of the marrow cavity on fluid-sensitive sequences. Depending on etiology, histology can show interstitial hemorrhage, organizing granulation tissue, necrosis, fibrosis, cellular infiltrate, or reparative microcallus [29, 35, 36]. At microscopy, actual BME (extracellular fluid) is rarely seen. Alternative terms include BME pattern, BME-like signal, and edema-like marrow signal [31, 35, 36]. Elsewhere in this Glossary, BME is replaced with BME-like signal.

#### Imaging

Fat-suppressed T2W MR images are most sensitive for BME. On T1W images, marrow fat is effaced but not entirely replaced, differentiating BME from malignancy. BME has a similar MR appearance in traumatic, degenerative, inflammatory, neoplastic, ischemic, and idiopathic conditions. BME location and clinical scenario together guide differential diagnosis. In traumatic injury or overuse activity, subchondral BME reflects mechanical overload (e.g., bone bruise, stress response). In spondyloarthropathy, SAPHO (synovitis, acne, pustulosis, hyperostosis, and osteitis), and CRMO (chronic recurrent multifocal osteomyelitis), BME reflects inflammation (e.g., osteitis, enthesitis). Dual-energy CT can demonstrate BME using three-material decomposition algorithms [37, 38] (see also “Bone bruise,” “Osteitis condensans ilii,” “Stress response,” “Subchondral insufficiency fracture,” “Transient osteoporosis”).

### Bone mineral density

#### Pathogenesis

Bone mineral density (BMD) is a surrogate marker of bone strength. Lower BMD correlates with reduced bone strength and increased fracture risk [39]. Dual X-ray absorptiometry (DXA) is a standardized technique for measuring BMD [40]. Based on BMD and clinical risk factors (e.g., glucocorticoid exposure, cigarette smoking, low body mass index (BMI)), patients may be treated pharmacologically to mitigate fracture risk. FRAX (Fracture Risk Assessment Tool) can guide medical therapy by calculating a 10-year probability of osteoporotic fracture based on the integration of BMD with clinical risk factors (e.g., previous fracture, smoking history, glucocorticoids). Fragility (acute) fractures most commonly involve the hip, spine, pelvis, humerus, and wrist. Insufficiency (stress) fractures usually affect the spine, sacrum, and other weight-bearing bones. Longitudinal DXA monitors BMD and treatment response.

#### Imaging

DXA BMD of the spine and hip are interpreted using the *T* score definition of osteoporosis and osteopenia [40, 41]. A *T* score compares the BMD of a patient to a young, healthy reference population matched for gender and ethnicity. In postmenopausal women, osteoporosis is defined as a *T* score < − 2.5 standard deviations below mean BMD. A *T* score ≥ − 1 standard deviations is normal BMD. Osteopenia is diagnosed when the T score is between − 1 and − 2.5 standard deviations below mean BMD. In children, young adults, premenopausal women, and men under 50 years, *Z* scores are used to compare the BMD of a patient to a reference population matched for gender, age, and ethnicity. DXA has limitations [41]. It does not take into account bone quality (trabecular structure). DXA BMD may be normal despite the diagnosis of osteoporosis due to fragility fracture. As a 2D technique, DXA measures BMD in grams of mineral per square centimeter. Quantitative CT has the advantage of volumetric measurement (milligrams per cubic centimeter) [42]. It can also isolate trabecular bone, which is more sensitive to change due to disease progression or treatment. However, quantitative CT is limited by a lack of population-based reference data and requires a higher radiation dose (see also “Osteoporosis,” “Pathologic fracture”).

### Boutonniere deformity

#### Pathogenesis

Boutonniere deformity is pathologic flexion of the proximal interphalangeal (PIP) and hyperextension of the distal interphalangeal (DIP) joints of a finger. The alignment abnormalities are clinically obvious and specific for lesions of the extensor mechanism. Extensor digitorum communis trifurcates into a single central slip that attaches to the dorsal base of the middle phalanx and two lateral slips that reunite before attaching to the dorsal base of the distal phalanx. The primary disorder is stretching or tear of the central tendon slip causing PIP flexion. Progressive PIP flexion further disrupts the extensor retinaculum allowing the proximal phalanx to become “buttonholed” between the lateral bands. DIP extension is secondary. The boutonniere deformity occurs in inflammatory arthritis and traumatic tendon laceration [43].

#### Imaging

Radiographs show PIP flexion and DIP hyperextension. Associated findings may include inflammatory arthropathy or volar plate fracture of the middle phalangeal base. MR demonstrates synovitis and tenosynovitis in inflammatory arthritis. In trauma, MR delineates the central tendon slip, extent of tear, and degree of retraction (see also “Swan neck deformity”).

### Brodie’s abscess

#### Pathogenesis

Brodie’s abscess represents an intraosseous cavity of pus walled off by granulation tissue, reactive sclerosis, and inflammation [44]. It occurs in subacute and chronic osteomyelitis. It predominates in children and young adults. The most common organism is *Staphylococcus aureus*, but cultures are often sterile. Clinical presentation and diagnosis can be delayed due to nonspecific symptoms, afebrile body temperature, and normal serum inflammatory markers (e.g., erythrocyte sedimentation rate, C-reactive protein).

#### Imaging

Most cases involve the femur or tibia. Metaphyseal involvement is twice as common as diaphyseal. Radiographs classically show a rounded or serpentine lucency bounded by a sclerotic rim that fades gradually into surrounding cancellous bone. On MR, the abscess is fluid-like and nonenhancing. A characteristic MR feature is an inner ring of increased T1W signal intensity (penumbra sign) corresponding to granulation tissue [45]. A concentric, low-signal outer ring represents the sclerotic rim. Surrounding edema and inflammation show contrast enhancement. The differential diagnosis includes eosinophilic granuloma, osteoid osteoma, sarcoma, and metastasis.

### Buford complex

#### Anatomy

The Buford complex is a developmental variation referring to the combination of thickened, cord-like middle glenohumeral ligament (MGL) and absent anterosuperior labrum [46]. It occurs in approximately 1.5% of shoulders [46]. Anatomic variations of the capsulolabral complex are common in the anterosuperior quadrant of the glenoid rim. At arthroscopy, the normal anterosuperior labrum can be firmly attached to the glenoid rim, partially undercut by a sublabral sulcus or completely undercut by a sublabral foramen. The normal MGL is usually sheet-like, but it can be cord-like, thread-like, or absent. The cord-like MGL resembles the biceps tendon in caliber [46]. The anterosuperior labrum and MGL tend to have an inverse size relationship. The thicker the MGL, the smaller the anterosuperior labrum.

#### Imaging

On MR, the Buford MGL is thickened and cord-like but normal in location between the glenoid rim and subscapularis tendon [47]. The anterosuperior glenoid rim is bare and uncovered by labrum. The Buford complex is important to recognize because the thickened MGL can be mistaken for an avulsed, displaced labral fragment [46]. These diagnostic possibilities are distinguished based on the course of the structure. Distally, the MGL merges with the subscapularis tendon, whereas the torn labral fragment reunites with the anteroinferior glenoid rim. The Buford complex is associated with symptomatic superior labral tear (SLAP tear) [46, 48] (see also “Shoulder, SLAP tear (superior labrum anterior-to-posterior)”).

### Cam deformity

See “Hip impingement, cam deformity.”

### Cancellous bone

#### Anatomy

Cancellous bone (trabecular bone, spongiosa) is a structural scaffolding critical in skeletal load-bearing. Cancellous bone comprises 20% of skeletal mass and is concentrated in tubular and short bones. In tubular bones, it predominates in the epiphysis and metaphysis and transmits force from the subchondral bone plate to the diaphyseal shaft. Its 3D architecture depends on physiological stress and follows the biomechanical principles described by Julius Wolff in 1892, referred to as Wolff’s law. Trabecular architecture contributes to bone strength and adapts to stress changes. Trabecular thickness (mineral density) also contributes to bone strength. Cancellous bone is more metabolically active than compact bone. In osteoporosis and metabolic disorders (e.g., anorexia nervosa), weakened cancellous bone is a major risk factor for fragility fracture.

#### Imaging

Cancellous bone is visible on radiographs, high-resolution CT and high-resolution MR. Patterns of compressive and tensile trabeculae are most distinctive in the proximal femur and calcaneus. On MR, signal intensity depends on the distribution of hematopoietic (red) and fatty (yellow) marrow. Whereas red marrow shows signal heterogeneity, yellow marrow has the uniform appearance of fat (see also “Bone mineral density,” “Osteoporosis,” “Wolff’s law”).

### Carpal boss

#### Pathogenesis

The carpal boss is a bony protuberance located dorsally at the second and/or third carpometacarpal junction [49]. It shows several different configurations. It usually fuses with the second or third metacarpal base. Less frequently, it fuses with the capitate or trapezoid. In approximately 25% of cases, it is completely unattached and, therefore, represents an accessory ossicle (os styloideum). The pathogenesis is unknown. Traumatic, degenerative, and developmental etiologies have been proposed. Carpal boss may be symptomatic (carpal boss syndrome) or asymptomatic [50]. Localized pain may result from mechanical friction with an adjacent bone or tendon.

#### Imaging

Radiographs, CT, MR, and US can confirm the diagnosis of suspected carpal boss or reveal an incidental, asymptomatic carpal boss. The differential diagnosis includes osteophyte, fractured osteophyte, and old fracture fragment. CT and MR best characterize its relationship to the carpometacarpal junction. They discriminate osseous fusion from os styloideum and distinguish pseudarthrosis formation from joint degeneration. On MR, BME-like signal may correlate with clinical symptoms [51]. MR and US can depict a ganglion or extensor carpi radialis brevis tenosynovitis.

### Carpet lesion

#### Pathogenesis

The carpet lesion refers to focal delamination of articular cartilage. During arthroscopic probing, it undulates like a carpet (carpet phenomenon, wave sign). Delamination occurs at the tidemark, the junction between uncalcified and calcified cartilage [52]. Along this cleavage plane, compression and shearing forces separate the cartilage from the underlying subchondral bone plate. The carpet lesion is often associated with cam-type femoroacetabular impingement. It involves the anterosuperior acetabulum and may propagate into the labrum without chondrolabral disruption or visible surface defect. Patients usually present with pain. Mechanical symptoms suggest full-thickness chondral tear with flap formation.

#### Imaging

On MR, carpet lesions are difficult to diagnose because chondral thickness and surface contour are normal [53]. Confident diagnosis requires the presence of fluid in the cleavage plane between articular cartilage and the subchondral bone plate. In highly congruous joints like the hip, however, fluid accumulation is limited by the close apposition of cartilage and bone. Traction MR may separate cartilage from bone and promote fluid accumulation. Subchondral BME-like signal raises the possibility of occult delamination. On arthrographic CT and MR, contrast can leak across full-thickness chondral tears into the cleavage plane (see also “Hip impingement, femoroacetabular (FAI),” “Hip impingement, cam deformity”).

### Chondromalacia

#### Histopathology

Chondromalacia is a pathological process affecting hyaline cartilage in individuals without osteoarthritis [54]. The pathogenesis remains unclear. At arthroscopy, the spectrum of chondral abnormalities includes softening, blistering, surface fibrillation, fissuring, fragmentation, and bone exposure [55]. Histologically, collagen fiber disorganization and reparative metaplasia are similar to findings in early osteoarthritis [56]. Symptoms may include crepitus and pain. The patella is most commonly involved. In severe chondromalacia patellae, treatment options include resurfacing arthroplasty.

#### Imaging

MR is valuable in diagnosing and grading chondral lesions. Grading systems are typically modeled after arthroscopic classifications. MR demonstrates higher-grade lesions but is less sensitive for chondral softening, swelling, and surface fibrillation [57]. Arthrographic CT and MR may improve the detection of lower-grade surface lesions. Subchondral BME-like signal suggests overlying full-thickness fissure or flap. In chondromalacia patellae, the trochlear cartilage is typically normal. In osteoarthritis, both the patella and trochlear cartilage are involved (see also “Osteochondral defect,” “Carpet lesion”).

### Compartment syndrome

#### Pathogenesis

Compartment syndrome results from elevated pressure and impaired circulation in a muscle compartment. Excessive intracompartmental pressure causes irreversible myonecrosis if it exceeds perfusion pressure, or reversible ischemia if does not. Etiologies include trauma, overexertion, infection, and neoplasm. The lower leg is more commonly affected than the thigh, forearm, and paraspinal compartments. The condition may be acute or chronic. Acute compartment syndrome is a surgical emergency requiring decompression and fasciotomy. Clinical diagnosis is based on intracompartmental pressure measurement. Chronic and exertional compartment syndromes are exacerbated by vigorous exercise.

#### Imaging

In both acute and chronic compartment syndromes, the value of imaging is unestablished. On MR and US, muscle edema and swelling are present with loss of normal fascicular architecture [58]. On contrast MR, enhancement is absent in myonecrosis and decreased in ischemia. In-scanner exercise can be used to diagnose chronic exertional compartment syndrome [59]. Chronic sequelae include muscle atrophy, scarring, and dystrophic calcification.

### Coracoacromial arch

#### Anatomy

The coracoacromial arch (CAA) is a bony and soft tissue roof that overlies the humeral head and rotator cuff. It comprises the coracoid process, coracoacromial ligament, acromion, and acromioclavicular joint. It forms the superior boundary of the supraspinatus outlet, the subacromial passageway for supraspinatus and infraspinatus between their scapular origins, and humeral insertions. Developmental, traumatic, and age-related abnormalities of the CAA are associated with outlet narrowing, subacromial impingement, and rotator cuff tear [60].

#### Imaging

Imaging enables subjective (e.g., acromial types) and quantitative (e.g., lateral acromial angle, acromiohumeral interval, critical shoulder angle) assessments of the CAA and supraspinatus outlet. On radiographs, anteroposterior, axillary, and outlet views demonstrate osseous spurs, os acromiale, acromial morphology, and osteoarthritis of the acromioclavicular joint. MR shows supraspinatus flattening or deformity, acromioclavicular joint BME-like signal, os acromiale pseudarthrosis, and subacromial–subdeltoid bursitis (see also “Shoulder, subacromial impingement”).

### Cortical bone

#### Anatomy

Mature bone consists of a cortical shell (compact bone, cortex) containing cancellous bone, the medullary cavity, hematopoietic (red) marrow, and fatty (yellow) marrow. Cortical bone is extremely dense, comprising 80% of total skeletal mass. It has an outer periosteal membrane and inner endosteum. At microscopy, the osteon, the anatomical and functional unit of cortical bone, forms a cylindrical column of concentric lamellae (layers of compact matrix) surrounding a central Haversian canal. The Haversian canal contains vessels supplying blood to the cellular elements of the osteon including osteoblasts, osteocytes, and osteoclasts.

#### Imaging

Cortical bone is densely mineralized on radiographs and CT. On MR, it appears as a signal void except on sequences employing ultrashort-TE. Pathological changes of cortical bone include periosteal reaction, saucerization, expansile remodeling, and bone destruction. Geographic, moth-eaten, and permeated patterns of bone destruction correspond to disease aggressiveness (see also “Cancellous bone,” “Periosteal reaction”).

### Crescent sign of osteonecrosis

#### Pathogenesis

The crescent sign refers to a subchondral fracture cleft in osteonecrosis. It immediately underlies the subchondral bone plate. It results from the mechanical failure of cancellous trabeculae, foreshadowing articular collapse, bone fragmentation, and secondary osteoarthritis [61]. Common locations include the femoral and humeral heads, followed by the scaphoid, lunate, and talus.

#### Imaging

Originally, the crescent sign was a radiographic finding [61]. Radiographs demonstrate a curvilinear subchondral lucency within a zone of necrotic bone. Flattening of the overlying subchondral bone plate indicates mechanical failure and imminent articular collapse [62]. CT is more sensitive for gas within the fracture cleft, suggesting subchondral bone plate motion or joint communication. On MR, fluid can fill the fracture cleft [31, 62]. The fracture may cross the subchondral bone plate and extend into the joint, creating an unstable osteochondral flap. In advance of subchondral fracture, BME-like signal distal to necrotic bone suggests stress response and imminent failure of subchondral trabeculae (see also “Double line sign in osteonecrosis”).

### Cyclops syndrome

#### Histopathology

Cyclops syndrome refers to postoperative decrease in knee extension following anterior cruciate ligament reconstruction [63]. Mechanical symptoms result from a nodular form of arthrofibrosis localized anteriorly in the intercondylar notch. The pathogenesis is related to graft impingement and repetitive microtrauma in knee extension. The nodule may cause pain and require arthroscopic resection. At arthroscopy, the mass-like, synovial-lined nodule shows surface vessels reminiscent of the eyeball of the mythological cyclops [63]. Histologically, the cyclops lesion shows organizing granulation tissue with variable hemorrhage, myxoid change, fibrosis, and cartilaginous or osseous debris. This tissue composition contrasts with the capsular scarring seen in generalized arthrofibrosis. The cyclops lesion also develops in nonoperated knees due to entrapment of anteriorly displaced ligament bundles following rupture or partial tear of the anterior cruciate ligament [64].

#### Imaging

On MR, both preoperative and postoperative cyclops lesions demonstrate heterogeneous T1W and T2W signal intensities and can measure several centimeters. On contrast MR, enhancement is variable. Symptomatic and asymptomatic lesions have similar MR appearances [65]. The differential diagnosis includes nodular synovitis and localized intra-articular tenosynovial giant cell tumor.

### Denervation myopathy

#### Pathogenesis

Muscle denervation results from partial or complete loss of nerve supply. It can be temporary or permanent and symptomatic or asymptomatic. Etiologies include neuropathy, autoimmune disorder, viral infection, prolonged nerve compression, nerve infiltration by neoplasm (carcinomatosis), and penetrating injury. Parsonage–Turner syndrome, also called idiopathic brachial neuritis, affects one or more muscles of the shoulder girdle [66]. Clinical diagnosis is challenging because pain and progressive weakness masquerade as more common disorders such as rotator cuff tear or capsulitis.

#### Imaging

In the acute stage of denervation, MR can show pathognomonic features such as diffuse muscle edema (edema-like signal), normal muscle bulk, and uniform contrast enhancement. In the subacute and chronic stages of denervation, findings are less specific including muscle atrophy, fat infiltration, and heterogeneous edema-like signal. Parsonage–Turner syndrome more commonly involves the supraspinatus and infraspinatus than deltoid, teres minor, and subscapularis muscles. MR may show causative abnormalities such as nerve encasement by tumor or discontinuity from laceration. Solid and cystic lesions may compress a neurovascular bundle (e.g., paralabral cyst in the spinoglenoid notch). MR neurography can shows signs of neuropathy, but findings are often negative or nonspecific.

### Dislocation

See “Subluxation.”

### Double line sign in osteonecrosis

#### Histopathology

The “double line sign” refers to a pathognomonic MR finding in osteonecrosis. Two adjacent serpentine lines demarcate the boundary between devitalized and viable bone marrow. Histologically, the outer line corresponds to reactive sclerosis [67]. The inner line corresponds to reparative granulation tissue [67, 68]. At this same boundary, creeping substitution represents the slow, centripetal, partial removal of necrotic marrow and its replacement by woven bone or newly formed trabeculae.

#### Imaging

On MR, all pulse sequences show the low-signal outer line at the interface between avascular and vascularized marrow. This uninterrupted line completely surrounds the infarcted region and corresponds to the serpentine rim of reactive sclerosis on CT [61]. On T2W MR images, the high-signal inner line is usually discontinuous and sometimes absent. It is most prominent in cancellous bone. The double line sign is seen on both fat-suppressed and nonfat-suppressed T2W images [61]. On nonfat-suppressed images, chemical shift can artifactually obscure or exaggerate the double line sign (see also “Crescent sign of osteonecrosis”).

### Enostosis

See “Bone island.”

### Enthesopathy

#### Pathogenesis

Enthesopathy refers to any pathologic process involving an enthesis. An enthesis functions to dissipate load at a site of ligamentous, tendinous, capsular, or fascial attachment to bone [69]. The enthesis organ integrates structures that function together. The Achilles enthesis, for example, incorporates the tendon, retrocalcaneal bursa, and bone. Therefore, Achilles enthesopathy encompasses tendinopathy, bursitis, erosive cyst-like changes, and osteitis. In enthesopathy, osteitis refers to secondary (reactive) osseous inflammation. The term enthesitis is reserved for rheumatological disorders that cause primary entheseal and osseous inflammation, hallmarks of spondyloarthropathy.

#### Imaging

Radiographs and CT depict chronic changes such as enthesophytes, erosions, calcifications, and hyperostosis. The tendon (or ligament) demonstrates fibrillar disruption and hypoechoic thickening on US, and hyperemia on color Doppler. Both US and MR can show soft tissue edema, bursitis, and osseous erosions. MR also demonstrates BME-like signal and osseous cyst-like changes. Enthesopathy and insertional tendinopathy share overlapping imaging features (see also “Epicondylitis,” “Reactive arthritis,” “Tendinopathy”).

### Epicondylitis

#### Histopathology

Epicondylitis refers to a clinical syndrome of pain, tenderness, and swelling over the humeral epicondyles. Causative factors include aging and overuse (e.g., tennis elbow, golfer’s elbow). Histology shows tendinopathy (tendinosis) and enthesopathy [70]. Collagen fiber disorganization and mucoid change are present with fibrosis and variable vascular proliferation [71, 72]. In epicondylitis, insertional tendon tears can propagate into the collateral ligament and lead to elbow instability.

#### Imaging

Radiographs may show calcific tendinopathy or ossific enthesopathy. US and MR are rarely performed for clinical diagnosis. In surgical candidates, they delineate the extent of tendon and ligament tear. These findings may guide surgical planning (e.g., debridement versus repair). US shows tendon hyperemia on color Doppler, enables dynamic maneuvers in suspected elbow instability, and guides percutaneous interventions. MR demonstrates epicondylar BME-like signal, muscle atrophy, and peritendinitis (see also “Enthesopathy,” “Tendinopathy”)*.*

### Femoral diaphyseal stress injury

#### Pathogenesis

Femoral diaphyseal stress injury refers to stress response or fracture involving the compressive (medial) side of the proximal femoral diaphysis [73]. It results from osseous overuse and repetitive axial loading. Patients present with exercise-related thigh or groin pain. It affects runners and military recruits almost exclusively. In early reports, the pathogenesis was incorrectly attributed to insertional enthesopathy and traction periostitis (similar to shin splints). Obsolete terms include adductor splints, thigh splints, and adductor insertion avulsion syndrome.

#### Imaging

In stress fracture, radiographs are positive for periosteal reaction and linear diaphyseal lucency. They may be negative in stress response. MR demonstrates focal periosteal and/or endosteal edema localized to the medial or posteromedial femoral cortex [74]. Intracortical signal suggests fracture regardless of globular or linear appearance [75] (see also “Stress response”).

### Femoroacetabular hip impingement

See “Hip impingement, femoroacetabular (FAI).”

### Friction syndrome

#### Pathogenesis

Friction syndrome is the painful chafing of structures due to repetitive rubbing and compression leading to localized inflammation. This overuse injury occurs where structures glide over bony eminences and compress interposed tissues, such as the iliotibial band where it slides over the greater trochanter or lateral femoral condyle. Friction syndromes involve the patellofemoral compartment (patellar tendon–lateral femoral condyle friction syndrome) and regions of tendon overlap (intersection syndrome).

#### Imaging

MR and US show edema, hyperemia, and inflammation in the region of friction and tissue compression. On contrast MR, bursal and adventitious bursal collections show surrounding enhancement. Osseous findings include BME-like signal acutely, and bony proliferative changes chronically (see also “Iliotibial band friction syndrome,” “Intersection syndrome”).

### Gamekeeper’s thumb

See “Skier’s thumb.”

### Geyser sign

#### Pathogenesis

The geyser sign refers to the arthrographic flow of injected contrast material from the glenohumeral joint across the acromioclavicular joint into a supraclavicular collection. The flow pattern can simulate the eruption of a geologic geyser. This phenomenon requires direct communication between the glenohumeral and acromioclavicular joints and, therefore, requires both full-thickness rotator cuff tear and acromioclavicular capsular tear. Patients may present with a slowly enlarging soft tissue mass over the shoulder.

#### Imaging

Radiographs show signs of chronic rotator cuff tear. The humeral head may abut the acromion or acromioclavicular joint. A focal, soft tissue mass-like opacity overlies the acromioclavicular joint. On arthrography and arthrographic CT or MR, contrast material fills the glenohumeral joint, subacromial–subdeltoid bursa, acromioclavicular joint, and supraclavicular collection [76, 77]. Contrast material may distend the acromioclavicular joint. Associated findings include massive rotator cuff tear. US-guided aspiration may temporarily relieve symptoms, but the collection usually recurs. Differential diagnosis includes cystic neoplasm.

### Glenohumeral instability

See “Shoulder, glenohumeral instability.”

### Glenoid retroversion

#### Pathogenesis

Glenoid retroversion refers to abnormal posterior angulation of the articular surface of the glenoid cavity. It may be developmental (e.g., dysplastic glenoid) or acquired (e.g., throwing athlete). Retroversion predisposes to posterior labral tear and posterior glenohumeral instability [76, 77]. In osteoarthritis, acquired retroversion results from the loss of posterior glenoid cartilage and bone stock. Unless corrected by anterior reaming or posterior augmentation, it is associated with poor outcomes following total shoulder arthroplasty.

#### Imaging

Glenoid version is measured on transverse CT or MR images. Using conventional 2D images, version is the angle between glenoid and scapular lines [78]. The glenoid line connects the anterior and posterior glenoid rim at mid-glenoid level. The scapular line connects the midpoint of the glenoid line with the medial tip of scapular blade. Neutral (normal) version approximates 0° relative to the scapular axis. CT 3D reconstructions can correct for variations in scapulothoracic orientation [79].

### Groin pain

#### Pathogenesis

The term “groin pain” replaces multiple previously used terms (e.g., athletic pubalgia, sports hernia, soccer groin). It encompasses a spectrum of diagnoses, including hip and spine disorders. In this glossary, the term is used more narrowly and refers only to adductor, pubis, and inguinal disorders. The adductor tendons, abdominal muscles, and inguinal fascia share a common pathophysiology. These structures are biomechanically related, converging on the pubis and merging with the symphyseal aponeurosis [79]. Overuse activities transfer load between them leading to predictable patterns of bone and soft tissue injury.

#### Imaging

MR usually depicts multiple related findings. The pubis can demonstrate osseous stress response. In chronic groin pain, the pubic symphysis shows degenerative change and alignment abnormality. Fluid clefts extend across the symphyseal capsule into the pre-pubic aponeurosis and origins of the adductor tendons (e.g., adductor longus) and/or rectus abdominis. These tears are associated with symphyseal instability and predispose to acute strain involving the dynamic myotendinous stabilizers around the pubis (see also “Stress response”).

### Haglund’s syndrome

#### Pathogenesis

Haglund’s deformity can lead to Haglund’s syndrome, which is a painful condition of the heel. The deformity refers to a prominent bony protuberance, or bursal projection, at the posterosuperior aspect of the calcaneus. It causes symptoms by irritating adjacent structures. The diagnosis of Haglund’s syndrome requires some combination of clinical symptoms, Haglund’s deformity, retrocalcaneal bursitis, supracalcaneal bursitis, and Achilles tendinosis. If conservative management fails, surgical treatments include calcaneal osteoplasty and osteotomy.

#### Imaging

In patients with clinically suspected Haglund’s syndrome, weight-bearing lateral radiographs best characterize the size and shape of the posterosuperior bony protuberance as well as its relationship to the Achilles tendon [80]. However, calcaneal morphology and angle measurements are nonspecific and correlate poorly with symptoms. Retrocalcaneal and pre-Achilles soft tissue thickening may be present. In symptomatic patients, US and MR show signs of deep retrocalcaneal bursitis and pre-Achilles edema or inflammation in addition to distal Achilles tendinosis, insertional tendinopathy, or partial tear. Calcaneal BME-like signal is present in extreme cases. US and fluoroscopy enable image-guided injection of the retrocalcaneal bursa.

### Heterotopic ossification

#### Histopathology

Heterotopic ossification (HO) refers to extraskeletal bone that is identical biologically and histologically to normal cortical and cancellous bone [81]. It can arise in muscle, tendon, ligament, and other soft tissues and cause painful decrease in movement. Surgical resection may be necessary to restore function. Etiologies are multifactorial. Myositis ossificans circumscripta is associated with traumatic muscle injury. Postoperative HO may complicate orthopedic surgeries such as arthroplasty and other hardware placement. Juxta-articular HO may develop in immobilized patients after severe burn and brain or spinal cord injury. Several rare genetic diseases cause diffuse, debilitating HO (e.g., fibrodysplasia ossificans progressiva).

#### Imaging

In mature HO, radiographs show a pathognomonic shell of cortical bone. On CT (or MR), the intramedullary space demonstrates fat attenuation (or fat signal intensity) similar to cancellous bone. In the absence of radiographs, MR alone may lead to the erroneous diagnosis of lipoma when the cortical shell is thin or misinterpreted as a pseudocapsule, and the mineralized trabeculae resemble septations of adipose tissue (see also “Myositis ossificans”).

### Hill–Sachs defect

#### Pathogenesis

The Hill–Sachs defect is a depressed fracture of the posterosuperior humeral head. It results from humeral head impaction against the glenoid rim and, therefore, is pathognomonic of anteroinferior glenohumeral dislocation. A small defect does not affect prognosis. Repeated dislocations lead to a larger defect that may engage the anterior glenoid rim when the shoulder is abducted and externally rotated. The engaging Hill–Sachs defect is associated with glenoid rim fracture, decreased glenoid bone stock, and chronic instability [82]. Surgical treatments include remplissage and packing with size-matched iliac autograft or humeral osteoarticular allograft.

#### Imaging

On radiographs, the anteroposterior internal rotation view best demonstrates the wedge-shaped Hill–Sachs defect. CT and MR can show smaller defects and mild cortical flattening at the level of the humeral head apex [83]. At the level of the anatomical neck, normal flattening can be misinterpreted as a Hill–Sachs fracture. In the absence of a Hill–Sachs defect, MR may reveal subcortical BME-like signal at the posterosuperior humeral head indicating acute or subacute injury and prompting closer inspection of the glenoid rim for subtle fracture or Bankart lesion.

### Hip impingement, cam deformity

#### Pathogenesis

Cam deformity refers to a morphologic abnormality of the femoral head and head–neck junction [84]. It may cause painful femoroacetabular contact (impingement) during hip flexion and internal rotation. The femoral head is abnormally eccentric (nonspherical) in shape due to a laterally increasing radius, or bump. In femoroacetabular impingement (FAI), the cam deformity creates shearing forces on the acetabular cartilage leading to chondral delamination (carpet lesion), chondrolabral tear and, ultimately, premature osteoarthritis of the hip. To eliminate the cause of impingement, surgical treatment includes femoral neck osteoplasty (femoroplasty, bumpectomy).

#### Imaging

On anteroposterior radiographs, the cam deformity creates a pistol grip appearance of the proximal femur. Femoral head sphericity and head–neck offset can be assessed on anteroposterior, frog-leg lateral, cross-table lateral, and 45° Dunn views. The Dunn view is most sensitive for contour abnormalities at the anterior femoral head–neck junction. On CT and MR, quantitative measurements include alpha angle, femoral offset, and femoral distance around the femoral head circumference [5, 85, 86]. However, these measurements are unreliable for discriminating asymptomatic individuals from FAI patients [85, 86]. CT 3D reconstructions enable surgical planning for osteoplasty and virtual simulations of treatment outcomes. MR and arthrographic MR demonstrate labral tears and chondral lesions [53] (see also “Carpet lesion,” “Hip impingement, femoroacetabular (FAI),” “Hip impingement, pincer deformity,” “Impingement syndrome”).

### Hip impingement, femoroacetabular (FAI)

#### Pathogenesis

FAI syndrome is a painful decrease in hip movement due to mechanical abutment between the femur and acetabular rim [87]. Abutment results from abnormal morphologies of the femoral head, acetabular rim, or both. On the femoral side, the cam deformity refers to abnormal sphericity of the femoral head and decreased offset of the head–neck junction. On the acetabular side, the pincer deformity refers to focal or diffuse overcoverage of the femoral head by the acetabular rim. FAI is associated with premature osteoarthritis [87]. Similar to other impingement syndromes, the diagnosis of FAI depends on both clinical and imaging evidence.

#### Imaging

Imaging studies support the clinical diagnosis of FAI syndrome and guide management [5]. Radiographs and CT show predisposing osseous abnormalities (cam and pincer lesions). However, similar morphologies may be present in asymptomatic patients [88]. MR and arthrographic MR demonstrate labral tears and chondral lesions [53]. Chondral damage is associated with the worst outcomes (see also “Hip impingement, cam deformity,” “Hip impingement, pincer deformity,” “Impingement syndrome,” “Acetabular retroversion”).

### Hip impingement, ischiofemoral (IFI)

#### Pathogenesis

IFI refers to symptomatic conflict between the lesser trochanter of femur and the ischial tuberosity of pelvis. Narrowing of the ischiofemoral space may entrap intervening structures (e.g., quadratus femoris muscle, common hamstring tendon) [89]. The narrowing is usually congenital and often bilateral. Acquired etiologies include intertrochanteric fracture, valgus osteotomy, exostosis, and soft tissue mass. Following total hip arthroplasty, IFI is associated with medial offset of the implant.

#### Imaging

In the acute setting, MR shows edema of quadratus femoris muscle and, less frequently, adventitious bursitis. In chronic cases, findings include fatty atrophy of quadratus femoris muscle, tear of common hamstring tendon, and osseous cyst-like lesions of ischium. On CT and MR, measurements may be difficult to standardize because the width of the ischiofemoral space depends on leg positioning (abduction versus adduction, internal versus external rotation, and flexion versus extension). Excessive external rotation, adduction, and extension overestimate the degree of narrowing [89].

### Hip impingement, pincer deformity

#### Pathogenesis

Pincer deformity refers to a morphologic abnormality of the acetabulum and acetabular rim [84]. It may cause painful femoroacetabular contact and movement restriction due to overcoverage of the femoral head. Overcoverage may be global in hips with a deep socket or focal in hips with acetabular retroversion or acetabular ossicle. In femoroacetabular impingement, pincer deformity leads to anterosuperior labral damage, chondrolabral tear, chondral defect and, occasionally, posteroinferior contrecoup lesion. To eliminate the cause of impingement, surgical treatment might include acetabular rim reconstruction (osteochondroplasty) or ossicle resection.

#### Imaging

Anteroposterior radiographs enable numerous assessments of acetabular coverage [90]. Coxa profunda and protrusio acetabuli are signs of global overcoverage. Tonnis angle, lateral center-edge angle, and acetabular index provide estimates of both under- and overcoverage. The crossover and ischial spine signs are most specific for acetabular retroversion and focal anterosuperior overcoverage. The posterior wall sign assesses prominence or deficiency of the posterior wall. CT and MR depict acetabular version and os acetabuli. CT 3D reconstructions enable surgical planning for osteochondroplasty and virtual simulations of treatment outcomes. MR and arthrographic MR characterize labral tears and chondral lesions [5] (see also “Hip impingement, femoroacetabular (FAI),” “Hip impingement, cam deformity,” “Acetabular retroversion”).

### Iliotibial band friction syndrome

#### Pathogenesis

The iliotibial band (ITB), or tract, is a thickening of the fascia lata over the lateral thigh. Friction syndromes occur where the ITB glides repeatedly over the greater trochanter of the hip or the lateral femoral condyle of the knee. During activity, the ITB slips over these bony eminences, rubbing and compressing interposed tissues. Exercise-related pain results from localized inflammation. Clinically, these overuse injuries are diagnosed most commonly in runners (runner’s knee) and cyclists. External snapping hip is a friction syndrome caused by the iliotibial band catching on the greater trochanter during hip motion and producing a painful clunking sensation or audible snap.

#### Imaging

MR and US show soft tissue edema and inflammation between the ITB and bone. At the hip level, trochanteric bursitis may be associated with tendinopathy or tear of the gluteus medius or minimus tendon [91]. At the knee level, adventitious bursitis shows surrounding enhancement on contrast MR. Acute osseous findings include BME-like signal. Chronic changes comprise soft tissue scarring and bony proliferation. In cases of external snapping hip, dynamic US can differentiate mechanical catching caused by the iliotibial band versus the iliopsoas tendon (see also “Friction syndrome,” “Trochanteric syndrome”).

### Impingement syndrome

#### Pathogenesis

Impingement syndrome refers to a painful decrease in joint motion resulting from the mechanical abutment of osseous and/or soft tissue structures. Developmental osseous anomalies may predispose to abutment. Overuse activity and trauma may lead to structural hypertrophy and deformities that produce abutment. Impingement syndromes are most common in the shoulder, hip, ankle, and wrist and are classified as internal or external. Internal and external impingements are caused by the abutment of intra-articular and extra-articular structures, respectively.

#### Imaging

Diagnostic criteria rely on clinical symptoms and signs. On physical examination, impingement tests reveal painful limited range of movement. Imaging studies support the clinical diagnosis and guide management, including surgical treatments. Radiographs and CT show predisposing osseous abnormalities. Dynamic US maneuvers may show structural abutment by soft tissue or bony abnormalities. US may also guide injection of local anesthetic and corticosteroid. MR adds detailed information about BME-like signal and soft tissue sequelae including labral tears, chondral defects, ligament injuries, and capsulitis (see also joint-specific impingements).

### Intersection syndrome

#### Pathogenesis

Intersection syndrome is an overuse disorder of the dorsal forearm where the tendons of the first extensor compartment (abductor pollicis longus and extensor pollicis brevis) cross over the tendons of the second extensor compartment (extensor carpi radialis longus and brevis). Localized pain and swelling result from repeated activity, friction, and secondary tenosynovitis. Intersection syndrome may also involve the dorsal wrist where the tendon of the third extensor compartment (extensor pollicis longus) crosses over the tendons of the second extensor compartment distal to Lister’s tubercle.

#### Imaging

MR and US may show fluid in tendon sheaths at the crossover point. Peritendinous edema and inflammation demonstrate enhancement on contrast MR and hyperemia on power Doppler [92]. The tendons appear normal in the absence of chronic stenosing tenosynovitis.

### Ischiofemoral hip impingement

See “Hip impingement, ischiofemoral (IFI).”

### Ivory vertebra

#### Pathogenesis

The ivory vertebra, or ivory vertebra sign, is a radiographic finding that refers to diffuse increase in opacity (attenuation) of a vertebral body without change in its size or contour. Both benign and malignant lesions may stimulate osteoblastic overproduction of mineral. In adults, common etiologies include osteoblastic metastasis (e.g., prostate, breast), lymphoma (usually Hodgkin lymphoma), SAPHO syndrome, and chronic infectious spondylitis. Rarely, ivory vertebra results from primary sarcoma, sarcoidosis, or systemic mastocytosis. Although Paget disease may increase vertebral attenuation, expansile bony remodeling falls outside the strict criteria for ivory vertebra sign.

#### Imaging

On radiographs and CT, the vertebral body is homogeneously or heterogeneously increased in attenuation with variable involvement of posterior elements [93]. On MR, decreased signal intensity is proportionate to the degree of sclerosis. Signs of malignancy include mixed osteolysis–sclerosis, loss of trabecular architecture, and extraosseous soft tissue extension. Paget’s disease involves the entire vertebra with bone enlargement, cortical thickening (“picture frame” appearance), coarsened trabeculae, and fatty marrow foci (see also “SAPHO syndrome”).

### Lisfranc joint

#### Anatomy

The Lisfranc joint comprises multiple articulations at the tarsometatarsal junction. Dorsal, interosseous, and plantar ligaments stabilize tarsometatarsal, intertarsal, and intermetatarsal articulations. Interosseous ligaments are strongest. The first intermetatarsal space is unique in that there is no interosseous ligament between the metatarsal bases. In lieu of this ligament, the Lisfranc ligament links the medial cuneiform to the second metatarsal base and thereby becomes a primary stabilizer of both the tarsometatarsal junction as well as the medial and intermediate columns of the foot [94]. Injury to the Lisfranc ligament may lead to tarsometatarsal instability, malalignment, and osteoarthritis.

#### Imaging

In high-energy trauma (e.g., motor vehicle accident), radiographs, and CT best characterize Lisfranc fracture–dislocation. In low-energy trauma (e.g., twisting injuries), MR best demonstrates Lisfranc ligamentous injury [95]. In suspected instability, weight-bearing radiography and CT reveal tarsometatarsal malalignment. Contralateral comparison improves the detection of subtle malalignment.

### Looser zone

#### Pathogenesis

Looser zones, also known as Milkman lines and pseudofractures, are hallmarks of osteomalacia. Looser, a surgeon, described radiolucent zones in patients with starvation after WWII. Milkman’s syndrome, named after the radiologist Louis Milkman, is a form of osteomalacia complicated by looser zones. Osteomalacia (rickets in children) is a metabolic disorder characterized by insufficient mineralization of osteoid matrix. A common cause of osteomalacia is vitamin D deficiency due to poor nutrition, inadequate sunlight exposure, malabsorption, or chronic disease (e.g., renal failure). Histologically, looser zones represent accumulations of unmineralized osteoid. In weight-bearing bones, they occur in the same locations as insufficiency fractures and, therefore, are often diagnosed and treated as fractures. In nonweight-bearing bones, they correspond to osteoid accumulation along nutrient foramina and, therefore, represent true pseudofractures [96].

#### Imaging

On radiographs, looser zones are linear, transverse lucencies with sclerotic margins oriented perpendicular to cortex [96]. They are often bilateral and symmetric measuring 2–5 mm in width. Common weight-bearing sites include pubic rami, medial femoral neck, and proximal femoral diaphysis. In the femur, in contrast with bisphosphonate-related fracture, they involve the medial, compressive-sided cortex rather than the lateral, tensile-sided cortex. Common nonweight-bearing sites include scapula, iliac wing, ulna, and ribs (see also “Osteoporosis,” “Bisphosphonate-related fracture”).

### Medial tibial stress syndrome

#### Pathogenesis

Shin splints is popular lay language for symptomatic conditions of the lower leg. In sports medicine, “medial tibial stress syndrome” (MTSS) refers specifically to exercise-related pain at the mid-distal tibial diaphysis. MTSS affects runners and overuse athletes engaged in intensive weight-bearing activities and represents stress response [97]. It falls along the continuum of tibial stress injury. Whereas MTSS patients may safely continue running at reduced mileage, stress fracture patients must discontinue running to avoid complications.

#### Imaging

Radiographs are often negative. On bone scintigraphy, longitudinal uptake may involve the compressive (posteromedial) side of the tibial cortex. CT may show focal regions of cortical bone resorption. On MR, periosteal and/or endosteal edema is present [76, 98]. CT and MR are valuable in excluding stress fracture and guiding rehabilitation (see also “Stress fracture,” “Femoral diaphyseal stress injury”).

### Metallosis

#### Pathogenesis

Metallosis refers to the tissue deposition of metallic wear debris. It results from erosive friction between pieces of metal. Repetitive motion and chronic hardware abrasion release microscopic metallic particles that accumulate in tissue and stain it darkly. It is associated with orthopedic hardware failure such as the fracture of a metal plate following internal fixation. Following arthroplasty, the term has been used to describe the gross deposition of metallic wear debris as well as a spectrum of biologic reactions to metal [99]. These reactions depend on implant design, metal composition, and patient-related factors such as innate susceptibility. In metal–metal (MoM) and modular implants, for example, adverse local or systemic reactions may result from bearing wear or trunnion corrosion.

#### Imaging

In metallosis, radiographs typically show signs of hardware failure. Adjacent soft tissues may demonstrate curvilinear and cloud-like opacities due to gross deposition of metallic wear debris. In metal–polyethylene hip and knee implants, these opacities are associated with abnormal metal–metal contact due to wear, fracture, or displacement of the polyethylene liner. Liner wear may also cause particle disease (periprosthetic osteolysis) due to the release of nonmetallic polyethylene debris. CT better delineates microdebris and complex, radiodense collections. On MR, metallosis collections are distinguished from simple postoperative seromas by decreased T2W signal intensity. T1W signal intensity can be increased compared to transudative fluid. Magnetic susceptibility (blooming) artifact depends on the concentration of metallic particles. Aspirated fluid is darkly discolored. Following hip replacement or resurfacing, tissue necrosis and mass-like tumors (pseudotumors) suggest adverse local tissue reaction (ALTR) (see also “Reaction to metal”).

### Mucoid change

#### Histopathology

Mucoid change commonly involves tendons, ligaments, and fibrocartilage. Predisposing factors include aging, mechanical stress, and/or traumatic injury. Initially, histological changes are adaptive and consist of cellular metaplasia with variable vascular proliferation. Ultimately, irreversible tissue degeneration results from the accumulation of connective tissue mucin and the disruption, disorganization, and disintegration of collagen fibers [72, 73]. Altered mesenchymal cells express a variety of cytokines and matrix molecules responsible for inflammatory phenomena [69]. Surgical treatment involves debridement of the mucoid tissue.

#### Imaging

On MR, mucinous regions show nonspecific linear or globular increase in T2W signal intensity. On US, the normal fibrillar architecture is replaced by hypoechoic foci. Power Doppler may demonstrate hyperemia surrounding the mucinous region. Mucoid degeneration is a common feature of chronic tendon disorders (e.g., lateral epicondylitis, rotator cuff tendinosis) [72]. Affected tendons (e.g., Achilles, quadriceps, biceps brachii) are predisposed to tear and spontaneous rupture [72, 100]. The ACL, the most commonly involved ligament, shows fusiform or focal mass-like thickening that may cause pain or mechanical symptoms without knee instability [101].

### Muscle injury

#### Pathogenesis

Muscle injury is a general term encompassing multiple traumatic mechanisms and anatomical sites. Mechanism classifications include strain (acute eccentric contraction), contusion (direct blow), and delayed-onset muscle soreness (vigorous overuse) [102]. Site classifications include muscle belly, myofascial junction, and myotendinous junction. Grading systems categorize the severity of injury (e.g., mild, moderate, severe) but have limited prognostic validity [103]. Terminology is controversial. Whereas “tear” is commonly used in communications between physicians, “strain” is preferred in communications with laypersons [103].

#### Imaging

Both US and MR have diagnostic roles in muscle evaluation. US enables a focused approach. It shows superficial injuries, permits dynamic maneuvers, and guides interventions. MR enables a global approach, depicting deep muscular compartments as well as bone marrow and joints. MR helps in tissue characterization (hematoma versus fluid) and differentiation of acute from remote injury [104]. Both US and MR enable the grading of strains as mild, moderate (partial tear), and severe (subtotal tear or rupture) (see also “Myotendinous junction,” “Myotendinous unit”).

### Myonecrosis

#### Pathogenesis

Myonecrosis refers to infarction of skeletal muscle. The most common etiologies include trauma (crush injury), compartment syndrome, prolonged immobilization, poorly controlled diabetes, radiotherapy, sickle cell crisis, or toxin (snake bite) [105]. Myoglobinemia (rhabdomyolysis) is a complication of acute muscle infarction. Nonspecific symptoms may delay clinical diagnosis. Calcific myonecrosis represents a chronic complication of posttraumatic compartment syndrome and usually involves the anterior compartment of the lower leg. Muscle necrosis, cystic degeneration, and recurrent hemorrhage lead to dense, dystrophic calcification.

#### Imaging

On contrast MR, nonenhancing muscle is pathognomonic of myonecrosis. Whereas infarcted tissue is completely avascular, punctate and curvilinear foci of enhancement suggest residual viable tissue [106]. In the subacute setting, rim enhancement indicates early healing but can simulate a phlegmon or abscess. Contrast injection is contraindicated in rhabdomyolysis. Noncontrast MR usually shows nonspecific muscle swelling and edema. Heterogeneous signal intensity suggests a combination of necrosis, hemorrhage, and liquefaction [107]. Chronic sequelae include muscle atrophy, scarring, and dystrophic calcification. In calcific myonecrosis, radiographs and CT show amorphous calcifications at the periphery of a fusiform mass. On MR, the central region demonstrates heterogeneous fluid in contrast to the fatty marrow of myositis ossificans (see also “Compartment syndrome”).

### Myopathy

#### Pathogenesis

Myopathy refers to the heterogeneous spectrum of diseases causing skeletal muscle fiber dysfunction. Examples include hereditary myopathies caused by enzymatic deficiencies and inborn errors of metabolism (e.g., glycogen storage disease). Idiopathic inflammatory myopathies encompass polymyositis, dermatomyositis, inclusion body myositis, and necrotizing autoimmune myopathy. Endocrine myopathies are associated with adrenal, thyroid, parathyroid, and pituitary dysfunction. Myopathies may be drug-induced (e.g., corticosteroid) or related to infection (e.g., HIV) and systemic conditions (e.g., sarcoidosis). Muscle biopsy is the diagnostic gold standard.

#### Imaging

Radiographs and CT demonstrate muscle mineralization and ossification. On MR, muscle edema, fat infiltration, and atrophy are related to disease activity, severity, and chronicity [105]. By depicting appendicular and axial muscle, whole-body MR can estimate the extent and distribution of myositis as well as treatment response. Patterns of muscle involvement may suggest particular myopathies [108]. Biopsy accuracy is improved by targeting areas of active disease (edematous, enhancing muscle).

### Myositis

#### Histopathology

Myositis is inflammation of muscle. Inflammatory myopathies are idiopathic, immune-mediated disorders that include dermatomyositis, polymyositis, inclusion body myositis, and syndromes overlapping with systemic rheumatological diseases. They may exhibit distinctive clinical and histopathological features, as in eosinophilic and granulomatous (e.g., sarcoid) myositis. In inflammatory myopathy, blind biopsy usually samples the quadriceps or deltoid muscle. Viral myositis may be complicated by rhabdomyolysis. Statin-related (drug-induced) myositis often persists after drug discontinuation. Bacterial myositis may be complicated by pyomyositis.

#### Imaging

Imaging may guide targeted biopsy. On STIR and fat-suppressed T2W images, MR shows the location and intensity of muscle edema [105, 109]. Inflamed muscle enhances on contrast MR. Whole-body MR depicts the distribution of myositis and overall disease activity. PET shows corresponding regions of increased muscle metabolism. On T1W images, fatty muscle replacement and atrophy indicate chronic (burned out) myositis. US demonstrates muscle edema, hyperemia (power Doppler), and perfusion (contrast-enhanced US) [110]. Differential diagnosis includes infection and pyomyositis.

### Myositis ossificans

#### Histopathology

Myositis ossificans (MO), the most common form of heterotopic ossification, is closely associated with trauma [79]. Synonyms include myositis ossificans circumscripta and myositis ossificans traumatica. MO has three histopathological stages [111]. During the first 3–4 weeks, tissue damage leads to organizing granulation tissue with fibroblastic and osteoblastic differentiation and osteoid formation. During the second 3–4 weeks, mineralized osteoid matrix develops with immature lamellar bone. Finally, after 8–10 weeks, immature bone evolves into mature lamellar cortical and trabecular bone.

#### Imaging

Imaging findings evolve with histopathological stage. Initially, radiographs are negative. MR features depend on the extent of myonecrosis and inflammation [111–113]. In earlier stages, enhancing, mass-like abnormalities can simulate sarcoma and, if biopsied, lead to misdiagnosis (pseudomalignant MO) [111, 112]. On radiographs and CT, intermediate stages show a zonal pattern of peripheral mineralization. Mature MO demonstrates a pathognomonic ossific shell that is better depicted by radiographs and CT than MR (see also “Heterotopic ossification”).

### Myotendinous junction

#### Anatomy

The myotendinous junction (MTJ), the most commonly injured component of the myotendinous unit, transmits tensile force between muscle and tendon. Terminal myocytes interdigitate with tendon fibers across deep folds and finger-like projections that increase contact area and dissipate energy during muscle contraction [114]. Despite this specialized interface, the MTJ is susceptible to viscoelastic failure and strain [115]. Muscles at greatest risk for strain cross two joints and generate extreme tensile forces (e.g., quadratus femoris, biceps femoris, biceps brachii, gastrocnemius) [102].

#### Imaging

In MTJ injury, comprehensive grading systems combine clinical and imaging findings to better estimate prognosis, guide rehabilitation, and predict return-to-play [103]. On US and MR, mild (low-grade) strain shows a feathery pattern of interstitial edema and hemorrhage centered around the MTJ. Moderate strain is associated with intramuscular hematoma and perifascial fluid. In severe (high-grade) strain, MTJ disruption results in tendon laxity or retraction. Signs of remote strain include old blood products, scar-like tissue, and fatty muscle atrophy (see also “Muscle injury,” “Myotendinous unit”).

### Myotendinous unit

#### Anatomy

The myotendinous unit, also known as the muscle–tendon–bone or muscle–tendon–enthesis unit, generates skeletal movement. Although it classically has five components (bone, enthesis, tendon, myotendinous junction, and muscle), normal variations exist. A tendon may attach to bone without an enthesis, and a muscle may attach to bone without a tendon. The proximal and distal osseous attachments are designated origin and insertion, respectively. Many factors influence failure location including patient age, traumatic mechanism, drug therapy, and systemic disease.

#### Imaging

On radiographs, the normal enthesis shows smooth cortical contour without cyst-like change or bony proliferation. On US and MR, the homogeneous fibrillar pattern of a normal tendon can artifactually resemble degeneration or tear due to anisotropy (US) or magic angle phenomenon (MR) [116]. Normal muscle shows a feathery, fascicular pattern and sharp interface at the myotendinous and myofascial junctions [104] (see also “Muscle injury,” “Enthesopathy,” “Myotendinous junction”).

### Necrotizing fasciitis

#### Pathogenesis

Necrotizing fasciitis is an aggressive bacterial infection involving subcutaneous fat and deep fascial compartments. Whereas non-necrotizing cellulitis and fasciitis are treatable by antibiotics, necrotizing fasciitis is caused by toxin-producing organisms that spread rapidly with infarction and liquefaction of fat and muscle. Early clinical signs and symptoms are nonspecific but may swiftly progress to limb discoloration, systemic toxicity, and sepsis. It is associated with fatal outcome unless diagnosed quickly and treated surgically with fasciotomy, debridement, or amputation [117].

#### Imaging

In emergent evaluation, CT can be obtained quickly. Although findings can be negative, CT is the modality most sensitive for gas. Soft tissue gas is a specific imaging finding, but the absence of gas does not exclude necrotizing fasciitis. Contrast-enhanced CT and MR show myonecrosis and liquefactive tissue infarction [118]. However, contrast injection may be contraindicated in patients presenting with rhabdomyolysis or renal failure. Other CT, MR, and US features may be seen in both necrotizing and non-necrotizing fasciitis, including dermal thickening, subcutaneous edema, muscle edema, fascial enhancement, and intracompartmental fluid collections [119] (see also “Myonecrosis”).

### O’Donoghue’s triad

#### Pathogenesis

O’Donoghue’s triad (unhappy medial triad) refers to trauma-related injuries involving the ACL, medial collateral ligament (MCL), and medial meniscus [120]. The triad is associated with both contact and noncontact (pivot shift) mechanisms incorporating abduction–external rotation of the knee [121]. Originally, diagnoses were based on open surgical inspection. In arthroscopic studies, the more common triad combines ACL, MCL, and lateral meniscal tear [121]. Coexisting lateral and medial meniscal tears may be present (unhappy medial tetrad).

#### Imaging

MR accurately depicts multi-ligament injury, meniscal tear, and chondral defect [122]. In surgical candidates, it allows the grading of cruciate and collateral ligament tears. It guides preoperative planning for ACL reconstruction, MCL repair, meniscal resection or repair, and chondroplasty. In the acute setting, MR depicts marrow contusions and fractures that elucidate traumatic mechanism and direct the targeted search for functionally related injuries such as the ramp lesion and lateral meniscocapsular tear (see also “Ramp lesion”).

### Osteitis condensans ilii

#### Pathogenesis

Osteitis condensans ilii refers to the benign, dense, localized sclerosis of iliac bones adjacent to sacroiliac joints. The etiology is unknown, possibly reflecting mechanical imbalance and chronic stress response [123]. Most patients are postpartum females in their third and fourth decades [124]. Although it may be associated with lumbago, the axial low back pain is not centered over the iliac lesions.

#### Imaging

Osteitis condensans ilii is often diagnosed incidentally based on characteristic radiographic and CT findings. The ilii show bilateral, sharply demarcated, densely sclerotic regions abutting the sacroiliac joints. Sclerosis is usually symmetric and anterior in location at the level of the middle thirds of the sacroiliac joints. In contrast to sacroiliitis, the sacroiliac joints are normal or show degenerative changes including vacuum phenomenon. CT may show mild sclerosis on the sacral side of the joint. On MR, mild BME-like signal is possible. On bone scintigraphy, radiotracer uptake can be increased. Findings may regress partially or completely over decades [124]. While the diagnosis is usually straightforward, the differential includes sacroiliitis and sacroiliac osteoarthritis in some cases.

### Osteochondral defect

#### Pathogenesis

Osteochondral defect is a nonspecific, focal lesion that involves articular cartilage, subchondral bone plate, and subchondral cancellous bone. This general term does not take into account onset (acute, subacute, chronic) or etiology (e.g., trauma, overuse, osteoarthritis) [31]. Whenever feasible, a more specific diagnosis should be made (e.g., osteochondral fracture, osteochondritis dissecans, articular collapse). Lesions isolated to cartilage (e.g., chondral delamination) or bone (e.g., subchondral insufficiency fracture) are not classified as osteochondral defects.

#### Imaging

On radiographs and CT, the subchondral bone plate is interrupted or depressed. A trabecular fracture cleft may be present. Chronic lesions may show fragmentation, sclerosis, and osseous cyst-like change. An unstable osteochondral fragment may be partially or completely surrounded by fluid or displaced from parent bone. MR adds diagnostic sensitivity by demonstrating chondral involvement and subchondral BME-like signal. On arthrographic CT and MR, contrast may flow between the osteochondral fragment and the parent bone (see also “Osteochondritis dissecans,” “Subchondral insufficiency fracture”).

### Osteochondritis dissecans

#### Histopathology

Osteochondritis dissecans (OCD) occurs in children and young adults. Typical locations include the medial femoral condyle and capitellum. Pathogenesis, natural history, and management are controversial. Histopathology is variable. Initially, subchondral bone shows organizing granulation tissue and fibrocartilagenous metaplasia. Trabeculae can demonstrate both necrotic and viable regions with osseous debris [125]. Perilesional clefts may coalesce into fissures leading to partial or complete detachment of the lesion from parent bone [125]. Unstable fragments usually fail conservative treatment and require surgical management.

#### Imaging

Radiographic and CT features depend on OCD location, chronicity, and stability. Perilesional lucency and sclerosis usually circumscribe the lesion [126]. Complications include fragmentation, collapse, and displacement [126]. MR can reveal intact articular cartilage despite subchondral bone plate disruption. After intravenous contrast, OCD enhancement indicates viability. The lack of enhancement signifies necrosis. An important MR sign of instability is fluid at the OCD interface with parent bone although granulation tissue may have a similar appearance [127]. Cyst-like changes and BME-like signal are less specific for instability [127]. At CT and MR arthrography, unstable fragments may be partially or completely surrounded by contrast.

### Osteonecrosis

#### Pathogenesis

Osteonecrosis is infarction of cancellous or cortical bone. It results from any condition causing irreversible ischemic death of bone cells (e.g., osteoclasts, osteoblasts, and osteocytes) and marrow cells (e.g., adipocytes, hematopoietic stem cells) [62]. Epiphyseal involvement may lead to important complications such as articular collapse [31]. In epiphyseal and subarticular locations, osteonecrosis was previously termed “avascular necrosis,” “aseptic necrosis,” or “ischemic necrosis.” In metaphyseal and diaphyseal locations, it was termed “bone infarction.”

#### Imaging

Radiographs and CT are negative or nonspecific in early osteonecrosis. Later findings include patchy sclerosis and rim calcification. MR demonstrates a pathognomonic, serpentine line at the interface of devitalized and vascularized bone. In epiphyseal osteonecrosis, the necrotic segment and serpentine line typically extend to the subchondral bone plate, thus differentiating it from subchondral fracture. On contrast MR, infarcted marrow is nonenhancing and easily discriminated from enhancing viable marrow. In metaphyseal and diaphyseal locations, osteomyelitis and skeletal tuberculosis are associated with sequestra (necrotic bone fragments) and sinus tracts [128]. In radiation necrosis, adjacent bone marrow appears edematous or fatty depending on the timespan following radiation treatment (see also “Crescent sign of osteonecrosis,” “Double line sign in osteonecrosis”).

### Osteoporosis

#### Pathogenesis

Osteoporosis is a systemic disease that decreases the skeletal strength of both compact bone and cancellous bone. Skeletal strength is determined by bone mass and structural quality. Bone mass is a measure of BMD, whereas structural quality is a measure of trabecular architecture and geometry. Osteoporosis causes low bone mass and structural weakness resulting in bone fragility and increased fracture risk. Fracture risk is usually assessed using BMD and categorized using dual X-ray absorptiometry (DXA). Standard categories consist of normal BMD (low fracture risk), osteopenic BMD (medium risk), and osteoporotic BMD (high risk). DXA screening identifies individuals who may benefit from drug treatment.

#### Imaging

Although radiographs and CT should not be used to diagnose osteoporosis, the skeleton may appear osteopenic (“washed out”). Both osteoporosis and osteomalacia cause osteopenia. In osteoporosis, bone mass is decreased with a normal ratio of mineral to matrix. In osteomalacia, deficient mineralization of osteoid results in a decreased ratio of mineral to matrix. Fragility fracture is an important sign of osteoporosis. DXA is the clinical standard for estimating bone mass and diagnosing osteoporosis [41]. It measures BMD in the lumbar spine, proximal femur, and distal radius. Quantitative CT measures BMD in the lumbar spine. Quantitative US assesses calcaneal bone quality. In research and clinical trials, advanced techniques (e.g., high-resolution peripheral quantitative CT) are used to evaluate bone quality [129] (see also “Bone mineral density,” “Looser zone”).

### Paratenonitis

#### Histopathology

Paratenonitis involves the membranous lining of a tendon that does not possess a synovial sheath (e.g., Achilles, patellar, and gluteal tendons). It is comparable to tenosynovitis in a tendon with a synovial sheath [130]. A common causative factor is overuse injury in athletes. The normally thin membrane of the paratenon provides a gliding mechanism with adjacent tissues. In early stages, mesenchymal cells and histiocytes proliferate and produce increased connective tissue mucin. Cytokine expression causes swelling and inflammatory phenomena [73, 130]. In chronic stages, the paratenon hypertrophies from fibrinous exudate and perivascular infiltrate [131, 132]. Peritendinous adhesions eventually restrict tendon movement.

#### Imaging

On high-resolution US and MR, the normal paratenon may be visible as a thin membrane that completely or partially surrounds a tendon [132]. In paratenonitis, US shows linear hypoechogenicity and hyperemia along the tendon border. The tendon itself is usually normal in appearance. Acutely, MR demonstrates a thin layer of fluid or edema in or around the paratenon [132]. Bursal fluid may be present. In chronic paratenonitis, edema and scar-like tissue extend into peritendinous fat (see also “Tendinopathy”).

### Pathologic fracture

#### Pathogenesis

Pathologic fracture occurs at the site of weakened bone due to preexisting abnormality. It happens spontaneously or results from minor trauma insufficient to fracture normal bone. The most common etiology is neoplasm, including metastasis, benign bone tumor (e.g., enchondroma, unicameral cyst, giant cell tumor), and primary bone malignancy (e.g., osteosarcoma, myeloma). Although osteoporosis weakens bone, osteoporotic fractures are not categorized as pathologic fractures. Fragility fracture is an acute fracture of osteoporotic bone caused by a single, low-energy traumatic event [133]. Insufficiency fracture is caused by cumulative load in osteoporotic bone and therefore is categorized as a stress fracture.

#### Imaging

On radiographs, fracture fragments can obscure small lesions and make it difficult to differentiate pathologic from nonpathologic fracture. During normal healing, cortical resorption can simulate a lytic lesion and lead to the false diagnosis of pathologic fracture. On CT and MR, soft tissue mass, focal marrow replacement, and distant lesions increase confidence in the diagnosis of pathologic fracture [134] (see also “Osteoporosis”).

### Periosteal reaction

#### Pathogenesis

The periosteum is a layered membrane that protects underlying bone, supplies nutrients, regulates growth, and guides remodeling. In development, it reacts to normal stimulants to support membranous ossification, endochondral ossification, and appositional bone growth. It reacts to abnormal stimulants by forming new bone in distinctive patterns. Patterns of periosteal reaction may help to distinguish aggressive from indolent conditions, and benign or systemic processes from malignancies. The following subtypes can help to narrow the differential diagnosis [135, 136].

#### Patterns of periosteal reaction

- Solid: mature bone formation suggesting a long-standing, indolent, benign process (e.g., osteoid osteoma, venous stasis, chronic osteomyelitis, hypertrophic pulmonary osteopathy)
- Single lamellar: a single layer of new bone suggesting an acute or subacute benign process (e.g., stress fracture, acute osteomyelitis, Langerhans cell histiocytosis) although malignant processes are possible (e.g., leukemia, sarcoma)
- Multilamellar: concentric layers of new bone (onion-skin) suggesting a malignant process of intermediate aggressiveness, although benign processes are possible (e.g., acute osteomyelitis, Langerhans cell histiocytosis)
- Spiculated: new spicules of bone extend radially from cortex (sunburst or hair-on-end) indicating a fast-growing, aggressive malignancy (e.g., sarcoma)
- Interrupted: periosteal discontinuity (Codman’s triangle) indicating a rapidly growing malignancy that disrupts the periosteum, preventing mineralization and new bone formation

### Peritendinitis

See “Paratenonitis.”

### Pincer deformity hip impingement

See “Hip impingement, pincer deformity.”

### Plantar plate

#### Anatomy

The plantar plate links the metatarsal neck to the proximal phalangeal base. This strong fibrocartilaginous structure reinforces the joint capsule and stabilizes the metatarsophalangeal (MTP) joint. It is thin centrally, providing a gliding path for flexor tendons. The periphery is thicker and, in the first MTP joint, incorporates the sesamoids. Turf toe, originally described in football players, refers to sprain, partial tear, or rupture of the first MTP plantar plate [137]. In lesser toes, most tears involve the second MTP joint and are associated with chronic conditions, alignment abnormalities, metatarsalgia, and instability [138].

#### Imaging

MR and US demonstrate continuity of the normal plantar plate as it cradles the metatarsal head and attaches to the phalangeal base. Collateral ligaments merge with the plantar plate. Following acute trauma, localized edema suggests low-grade sprain or tear with healing potential. In severe or repeated trauma, discontinuity and proximal retraction of the plantar plate increase diagnostic confidence for high-grade chronic tear [139].

### Posterosuperior shoulder impingement

See “Shoulder, posterosuperior impingement.”

### Pseudarthrosis

#### Pathogenesis

Pseudarthrosis (false joint) generally refers to mobile fracture nonunion. A fibrous, pseudosynovial capsule surrounds the nonunion and fills with viscous fluid that simulates synovial fluid and creates the appearance of a joint. Pseudarthrosis is most common in fractures of the scaphoid waist, humeral diaphysis, clavicular diaphysis, distal tibial diaphysis, and talar neck. Congenital tibial pseudarthrosis is associated with type 1 neurofibromatosis [140]. Pseudarthrosis may complicate spinal fractures in ankylosing spondylitis and surgical bone graft following failed spinal fusion. Bertolotti’s syndrome refers to the association of transitional lumbosacral junction with back pain due to pseudarthrosis formation. In Bertolotti’s syndrome, unilateral or bilateral pseudarthrosis results from contact and motion between an enlarged transverse process and the sacrum or ilium.

#### Imaging

In long bones, radiographs show fracture deformity and lack of osseous bridging. The fractured bone ends are either atrophic, appearing tapered and osteopenic, or sclerotic and enlarged by mature, hypertrophic callus. MR demonstrates fluid contained by a pseudosynovial capsule. In fibrous nonunion, scar-like tissue bridges the fracture margins instead of fluid. CT reformats best depict pseudarthrosis margins in irregular bones and bone graft discontinuity in the spine [141]. Single-photon emission computed tomography may be useful in equivocal diagnoses. In Bertolotti’s syndrome, the pseudarthrosis often resembles osteoarthritis including effusion, sclerosis, and cyst-like change.

### Pseudofracture

See “Looser zone.”

### Quadrilateral space syndrome

#### Anatomy

The quadrilateral space is bounded by teres minor superiorly, teres major inferiorly, triceps (long head) medially, and humerus laterally. The axillary nerve and the posterior humeral circumflex artery pass through this narrow space. Quadrilateral space syndrome results from neurovascular compression leading to vague pain, localized tenderness, and axillary neuropathy [142]. Causes include hypertrophied muscle, fibrous bands, and soft tissue mass.

#### Imaging

The quadrilateral space often appears normal in patients with symptoms and signs of quadrilateral space syndrome [143]. On MR and US, fibrous bands are rarely visible. Soft tissue mass or dissecting paralabral cyst may displace the neurovascular bundle. MR may show nonspecific edema or fatty atrophy involving teres minor and posterior deltoid muscles. MR neurography findings are nonspecific.

### Ramp lesion

#### Pathogenesis

The ramp lesion is a peripheral, vertical, longitudinal tear involving either the posteromedial joint capsule or medial meniscus at the meniscocapsular junction [144]. It is trauma-related and associated with rupture of the ACL. The ramp lesion can be missed at arthroscopy because of its location in the posteromedial “blind spot.” Unstable lesions are repaired arthroscopically to treat pain, restore mechanical function, and delay osteoarthritis.

#### Imaging

On MR, the ramp lesion can be difficult to diagnose in the absence of posterior meniscocapsular tissue distraction, which is more likely to occur in knee flexion than extension. A specific finding is fluid separating the posterior horn of medial meniscus from the joint capsule. Peripheral, vertical meniscal tear may also be present. In the setting of acute ACL rupture, diagnostic sensitivity is increased when the posterior meniscocapsular junction shows localized edema and the posteromedial tibial plateau shows marrow contusion [144]. Following remote trauma, ramp lesions are easily overlooked due to healing and scarring.

### Reaction to metal

#### Pathogenesis

Reaction to metal refers to the scope of adverse tissue responses elicited by metal-containing arthroplasty implants [145]. Causative wear particles include metallic debris and corrosion products but not polyethylene and cement debris. Adverse reaction to metal debris (ARMD) is an umbrella term incorporating both local and systemic responses. Adverse local tissue reaction (ALTR) applies specifically to periprosthetic (local) tissue alterations such as necrosis, inflammation, and tumor-like masses (pseudotumors). Aseptic lymphocytic vasculitis–associated lesion (ALVAL) is a subtype of ALTR [146]. The terms ARMD and ALTR are usually applied in the setting of MoM implants or femoral components with modular stems. They cause adverse local or systemic reactions due to bearing wear or tribocorrosion. In modular implants, corrosion occurs at the head–neck (trunnionosis) and neck–stem interfaces [145, 147].

#### Imaging

US, CT, and MR can demonstrate periprosthetic pseudotumor. This mass-like lesion usually comprises both solid tissue and complex fluid and distends the trochanteric or iliopsoas bursa [145]. By applying metal artifact reduction parameters to limit image degradation, MR can better show periprosthetic osteolysis, solid- and fluid-based synovitis, layering debris, pseudocapsule distension, pseudocapsule dehiscence, and tendon rupture. On contrast MR, nonenhancing periprosthetic tissue indicates necrosis and cytotoxic reaction. Pseudotumor and tissue necrosis compromise prognosis and revision arthroplasty (see also “Metallosis”).

### Reactive arthritis

#### Pathogenesis

Reactive arthritis (formally called Reiter’s syndrome) refers to a postinfectious arthropathy that is monoarticular or oligoarticular. It often meets the clinical criteria for axial or peripheral spondyloarthropathy [148]. Symptoms begin within several weeks following gastrointestinal or genitourinary infection [149]. Typical organisms include *Yersinia*, *Salmonella*, *Shigella*, *Campylobacter*, and *Chlamydia*. The knee is most commonly affected followed by the ankle, small peripheral joints, and sacroiliac joints. Some patients develop enthesitis (especially calcaneus at attachment sites of Achilles tendon and plantar fascia), dactylitis, urethritis, and conjunctivitis. Diagnosis is based on clinical and laboratory findings.

#### Imaging

In chronic reactive arthritis (> 6 months), radiographs may show signs of inflammatory arthritis or sacroiliitis. In earlier stages, MR and US demonstrate nonspecific synovitis, asymmetric sacroiliitis, and calcaneal enthesitis. The differential diagnosis includes inflammatory arthritides and seronegative spondyloarthropathies (ankylosing spondylitis, psoriatric spondyloarthritis, enteropathic spondyloarthritis) (see also “Enthesopathy,” “Synovitis”).

### Rice bodies

#### Histopathology

Rice bodies have the visual appearance and size of polished grains of white rice. Histologically, they are composed of fibrin in various stages of organization. The pathogenesis is unclear and possibly multifactorial. Rice bodies may begin as a nidus of cellular debris or necrotic synovial tissue. Although they represent a nonspecific response to chronic synovial inflammation, they are most characteristic of rheumatoid arthritis and, less often, tuberculous arthritis [150]. They can involve joints, bursae, and tendon sheaths. They rarely cause mechanical symptoms.

#### Imaging

Radiographs and CT may show signs of inflammatory arthropathy (erosions, joint space narrowing), but are negative for rice bodies given nonmineralization. On US, homogeneously heterogeneous echogenicity suggests the presence of rice bodies. On MR, rice bodies can be difficult to diagnose when they are tightly packed, uniform in size, and iso-intense with effusion. They are avascular and, therefore, lack enhancement following intravenous contrast. The main differential consideration is synovial chondromatosis.

### Rotator cuff tear

See “Shoulder, rotator cuff tear (overview and partial and full thickness).”

### SAPHO syndrome

#### Histopathology

SAPHO syndrome represents a rare inflammatory disorder involving bone, joints, and skin. The acronym refers to synovitis, acne, pustulosis, hyperostosis, and osteitis. Although the etiology is considered to be autoinflammatory, *Cutibacterium acnes* infection may activate the process in some cases. Cultures are usually negative, and biopsy shows sterile, nonspecific osteitis without sequestrum formation [151]. SAPHO has been associated with axial spondyloarthropathy and CRMO, a childhood variant of chronic nonbacterial osteomyelitis (CNO) [152].

#### Imaging

In adults, commonly involved sites include the sternocostoclavicular complex followed by the spine and sacroiliac joints. Radiographs and CT demonstrate erosive costoclavicular ankylosis, osteosclerosis, and thick periosteal reaction of the clavicle and first rib. Bone scintigraphy shows the “bull’s head sign” (avid uptake of ^99m^Tc-methylene diphosphonate by the sternomanubrial and bilateral sternocostoclavicular regions). In polyostotic disease, bone scintigraphy, PET, and whole-body MR are valuable for skeletal screening to identify sites of active osteitis. MR can demonstrate early osteitis, synovitis, enthesitis, spondylodiscitis, and sacroiliitis. In children and adolescents, long bone metaphases are commonly involved. Differential diagnosis includes osteomyelitis and malignancy.

### Sarcopenia

#### Pathogenesis

Sarcopenia refers to decreased skeletal muscle mass and impaired muscle function. Along with osteopenia, sarcopenia is a surrogate marker of physical frailty and functional decline related to age (geriatric patients) or chronic illness (cancer patients). Progressive sarcopenia leads to repeated falls, multiple fractures, and increased mortality [153]. Measurements of body composition, including muscle mass, fatty muscle infiltration (myosteatosis), fat distribution (visceral versus subcutaneous), and bone mineral density, have correlated with prognosis and medical outcomes [153, 154].

#### Imaging

Although CT, MR, and whole-body DXA enable quantitative and semiquantitative measurements of body composition, clinical implementation has been challenging [153, 154]. Imaging protocols, measurement techniques, and cutoff values are not standardized or validated [155].

### Serous atrophy of bone marrow

#### Histopathology

Serous atrophy (gelatinous transformation) refers to a non-neoplastic bone marrow disorder resulting from severe illness, poor nutrition, and weight loss. Rather than representing a primary disorder of bone marrow, common causes include anorexia nervosa, cancer-related cachexia, and chronic diseases (kidney or heart failure, acquired immunodeficiency syndrome). Bone marrow biopsy shows atrophy of adipose cells, paucity of hematopoietic cells, and accumulation of extracellular mucinous substances. Patients may develop osteoporosis and present with insufficiency fractures [156].

#### Imaging

MR shows striking abnormalities of fat. Marrow, visceral, and subcutaneous fat demonstrate T1W signal decrease and T2W signal increase [156]. These features may lead to misinterpretation and the presumption of scanner malfunction. Marrow does not enhance after contrast administration, differentiating serous atrophy from diffuse infiltrative malignancy. Radiographs typically show severe soft tissue wasting. On CT, visceral and subcutaneous fat are nearly absent.

### Shin splints

See “Medial tibial stress syndrome.”

### Shoulder, glenohumeral instability

#### Pathogenesis

Glenohumeral instability is static or dynamic [157]. Static instability refers to glenohumeral malalignment at rest and can be diagnosed radiographically. Disorders causing static instability include chronic rotator cuff tear (superior glenohumeral subluxation) and severe osteoarthritis (posterior subluxation). Dynamic instability refers to glenohumeral malalignment during physiological movement or load-bearing and is diagnosed clinically. Dynamic anterior instability can be classified as traumatic (TUBS) or atraumatic (AMBRI) in etiology. In TUBS (traumatic, unidirectional, Bankart lesion, surgery), imaging typically demonstrates signs of trauma that support the diagnosis of anterior instability such as Hill–Sachs fracture or capsuloligamentous tear [157]. In AMBRI (atraumatic, multidirectional, bilateral instability, rehabilitation), imaging is often nonspecific despite profound hyperlaxity and voluntary dislocation.

#### Imaging

In anterior instability, important osseous and soft tissue abnormalities may be conspicuous or inconspicuous. Radiographs, CT, and MR depict the glenoid rim, glenoid rim fracture, and loss of glenoid bone stock. MR and arthrographic MR or CT demonstrate Bankart, ALPSA (anterior labroligamentous periosteal sleeve avulsion), GLAD (glenolabral articular disruption), and HAGL (humeral avulsion of anterior glenohumeral ligament) lesions [158, 159]. ABER (abduction–external rotation) improves detection of the Perthes lesion [158, 159]. In posterior instability, posterior glenoid abnormalities include labral tear, capsular stripping, chondral defect, and humeral translocation. Multidirectional instability is associated with circumferential labral tearing.

### Shoulder, posterosuperior impingement

#### Pathogenesis

Posterosuperior (internal) impingement syndrome of the glenohumeral joint results from painful contact between the rotator cuff tendon and the posterosuperior labrum when the shoulder is abducted and externally rotated (ABER). Repetitive, excessive contact leads to “kissing” lesions of the cuff and labrum [160]. Posterosuperior impingement typically occurs in overhead athletes such as tennis players and baseball pitchers.

#### Imaging

US, MR, and arthrographic CT show articular-sided surface fraying or tearing of the supraspinatus tendon posteriorly and infraspinatus anteriorly [161]. The PASTA (partial articular supraspinatus tendon avulsion) lesion refers to an articular-sided rim rent at the tendon insertion site (footprint) to greater tuberosity. In arthrographic MR, the ABER position may increase diagnostic sensitivity for articular-sided cuff tear. Labral blunting and tearing are located posterosuperiorly or both posterosuperiorly and superiorly. Osseous findings include cysts and BME-like signal at the posterosuperior humeral head [161] (see also “Impingement syndrome,” “Shoulder, rotator cuff tear (partial thickness),” “Shoulder, SLAP tear (superior labrum anterior-to-posterior)”).

### Shoulder, rotator cuff tear (full thickness)

#### Pathogenesis

Full-thickness rotator cuff tear refers to tendon discontinuity resulting in communication between the glenohumeral joint and subacromial–subdeltoid bursa. Cuff tears most commonly involve supraspinatus, infraspinatus, and subscapularis tendons. Treatment outcome and prognosis correlate with tear size and muscle damage. Size is a measure of width (anteroposterior dimension) and length (medial–lateral retraction from greater tuberosity). Muscle damage is a measure of fatty infiltration and atrophic volume loss. Tendon delamination impairs tendon quality and healing potential [162, 163].

#### Imaging

At arthrography, injected contrast flows across a full-thickness tear from the glenohumeral joint into the subacromial–subdeltoid bursa. US, MR, and arthrographic CT demonstrate tear size and muscle damage [164]. Tear patterns can affect treatment decisions [165]. Common patterns include crescent-shaped, L-shaped, and U-shaped tears. L-shaped tears involve the rotator interval and typically propagate into subscapularis tendon. A sign of tendon delamination is differential retraction of articular and bursal layers [162] (see also “Shoulder, rotator cuff tear (overview and partial thickness)”).

### Shoulder, rotator cuff tear (overview)

#### Pathogenesis

The rotator cuff comprises supraspinatus, infraspinatus, subscapularis, and teres minor. Degenerative, age-related (e.g., impingement-related) tears involve tendons at or near the greater tuberosity insertion site. Acute, trauma-related tears are often more proximal (e.g., myotendinous junction) in location. Prognosis and treatment decisions depend on tear depth (full or partial thickness), tear width (anteroposterior dimension), tear length (medial–lateral retraction), and muscle damage (fatty infiltration, atrophy). Cuff delamination negatively affects tendon quality and healing potential [162, 163]. No single classification system is accepted given continual advances in imaging and arthroscopy, and recognition of new tear types and subtypes [164, 165].

#### Imaging

MR and arthrographic MR enable global assessment of the shoulder including coracoacromial arch, subacromial–subdeltoid bursa, rotator cuff tendons and muscles, glenoid labrum, biceps tendon, and articular cartilage. On arthrographic CT, the bursa and bursal-sided tendon are difficult to assess unless contrast leaks into the subacromial–subdeltoid space [166]. US enables targeted evaluation of cuff tendons and muscles. US is feasible in the presence of bulk metallic hardware [164] (see also “Shoulder, rotator cuff tear (partial and full thickness)”).

### Shoulder, rotator cuff tear (partial thickness)

#### Pathogenesis

Partial-thickness rotator cuff tear refers to intrasubstance, bursal-sided or articular-sided tendon disruption [167]. Intrasubstance tear is “concealed” because the bursal and articular surfaces appear normal at arthroscopy. Bursal-sided tear is associated with subacromial (external) impingement [167]. Articular-sided tear occurs in overhead athletes with posterosuperior (internal) impingement. The PASTA lesion is an articular-sided rim rent at the tendon insertion site (footprint) to greater tuberosity [168]. The PAINT (partial articular tear with intratendinous extension) lesion is an articular-sided rim rent that dissects into the tendon substance and creates a plane of delamination. Delamination impairs tendon quality and healing potential [163].

#### Imaging

US and MR show bursal-sided, intrasubstance and articular-sided partial-thickness tears [164]. Arthrographic CT can demonstrate articular-sided tears. In arthrographic MR, ABER reveals the extent of intratendinous delamination. In ABER, the delamination defect, a potential space, can fill with contrast because the tendon becomes lax and redundant enabling separation of the bursal and articular layers (see also “Shoulder, rotator cuff tear (overview and full thickness),” “Shoulder, posterosuperior impingement, “Shoulder, subacromial impingement”).

### Shoulder, SLAP tear (superior labrum anterior-to-posterior)

#### Pathogenesis

SLAP tears involve the superior labrum and may also involve the biceps tendon, glenohumeral ligaments, and other labral quadrants. Four original types were described [169]. In SLAP I, the labrum shows free-margin fraying. In SLAP II, labral detachment occurs at the chondrolabral junction and undercuts the biceps anchor. In SLAP III, bucket handle labral tear spares biceps. In SLAP IV, bucket handle labral tear propagates into biceps. SLAP I tears are commonly degenerative and asymptomatic. Overhead athletes are predisposed to SLAP II tears [170]. SLAP II tears may be surgically repaired with anchor stabilization of the biceps tendon. Other SLAP lesions may be debrided with biceps tenotomy or tenodesis.

#### Imaging

MR demonstrates contour abnormalities and chondrolabral defects of the superior labrum [171]. In SLAP tears, findings that increase diagnostic confidence include labral displacement from the glenoid rim, paralabral cyst formation, and biceps tendon lesion. On arthrographic MR and CT, contrast material outlines the labrum and fills tears [171]. The sublabral sulcus (cleft, recess), a normal developmental variation, partially undercuts the superior labrum at the chondrolabral junction and can be mistaken for tear (see also “Shoulder, posterosuperior impingement”).

### Shoulder, subacromial impingement

#### Pathogenesis

Shoulder impingements are classified as external (subacromial, subcoracoid) and internal (posterosuperior). mon shoulder impingement, results from mechanical compression of the rotator cuff, provoking tendinopathy [172, 173]. Tendinopathy may progress to partial and full-thickness cuff tears. Etiologies are multifactorial. Primary causative factors include structural abnormalities of the coracoacromial arch and chronic bursitis. Secondary factors include functional impairments such as muscle imbalance, glenohumeral instability, and scapulothoracic dyskinesis.

#### Imaging

No imaging findings are pathognomonic [174]. On radiographs, acromial morphologies, degenerative changes, and bony proliferative changes are common in both symptomatic and asymptomatic patients. US can show rotator cuff tendinopathy and tears, but objective dynamic criteria of impingement are limited. At MR, supporting findings include supraspinatus flattening by the acromion or acromioclavicular joint, acromial spurring, thickening of the coracoacromial ligament, subacromial–subdeltoid bursitis, supraspinatus tendinopathy and other rotator cuff abnormalities (see also “Impingement syndrome,” “Shoulder, rotator cuff tear (overview)”).

### Skier’s thumb

#### Pathogenesis

Skier’s thumb is acute injury of the ulnar collateral ligament (UCL) of the first metacarpophalangeal joint. It results from forceful hyperabduction as may occur in skiing when the thumb is caught in the strap of the ski pole. UCL injuries, including rupture, partial tear, and avulsion fracture, usually occur distally at the UCL attachment site to phalangeal head. The Stener lesion prevents healing to bone because the ruptured UCL is retracted proximal to the adductor pollicis aponeurosis [175]. Stener lesion, joint instability, and displaced avulsion fracture are indications for surgical stabilization. Although skier’s thumb and gamekeeper’s thumb are often used interchangeably, gamekeeper’s thumb refers to an overuse injury due to chronic stress and repetitive trauma that gradually stretch and attenuate the UCL [176]. Gamekeeper’s thumb exists in the absence of trauma and was described in gamekeepers who manually sacrificed wounded rabbits as part of their occupation [177].

#### Imaging

Radiographs may show metacarpophalangeal malalignment and avulsion fracture. Stress radiographs are sensitive for UCL insufficiency and metacarpophalangeal instability [166]. MR and US characterize the location and grade of UCL tear. They demonstrate the degree of ligament retraction and, in Stener lesion, interposition of the adductor pollicis aponeurosis between the retracted UCL and phalangeal head [177, 178]. US enables dynamic assessment of metacarpophalangeal instability (see also “Sprain”).

### Sprain

#### Pathogenesis

Sprain refers to ligamentous and capsuloligamentous injury, whereas strain refers to myotendinous injury. In mild (grade 1) sprain, the ligament is stretched but minimally torn. The ligament heals completely and remains competent. In moderate (grade 2) sprain, ligament fibers are partially disrupted. The ligament is weakened after healing and predisposed to recurrent injury. In severe (grade 3) sprain, the ligament is ruptured. Rupture may lead to chronic joint instability. Ligament sprain is most common in the knee, ankle, and acromioclavicular joint.

#### Imaging

Sprains are diagnosed and graded using US or MR. Injury grading is more accurate prior to tissue healing. In acute injury, the degree of hemorrhage and effusion are usually proportionate to injury grade [179, 180]. Edema outlines the ligament, showing regions of attenuation and discontinuity. Subtotal tear (near-complete fiber disruption) and rupture may have identical imaging appearances and prognoses. In remote sprain, grading is made more difficult by ligamentous scarring and tissue remodeling. As a result of this healing process, imaging findings may simulate an intact ligament despite clinical symptoms and signs of ligamentous incompetence and joint instability.

### Sports hernia

See “Groin pain.”

### Stress fracture

#### Pathogenesis

Stress fracture is the failure of bone due to repetitive loading and cumulative microdamage. The load is insufficient for acute fracture, but it exceeds the capability of bone to heal itself. It represents the final stage of stress injury. Subclassifications include fatigue fracture (overuse activity in normal bone) and insufficiency fracture (normal activity in weakened bone). Fatigue fractures typically occur in healthy athletes, runners, and military recruits. Insufficiency fractures are associated with metabolic and nutritional disorders (e.g., osteoporosis, female athlete triad). Irradiation is a common cause of insufficiency fracture.

#### Imaging

Whereas fatigue fractures tend to affect weight-bearing compact bone (e.g., medial femoral neck, tibial diaphysis, metatarsal shaft), insufficiency fractures tend to affect cancellous bone (e.g., vertebral body, sacral ala, subchondral bone) [76, 181]. On radiographs and CT, cortical stress fracture corresponds to a rounded or linear intracortical lucency. Periosteal reaction may be present. Trabecular stress fracture shows a sclerotic line that rarely intersects cortex. On MR, cortical stress fracture is associated with adjacent periosteal and endosteal edema. Trabecular stress fracture shows a low-signal line oriented perpendicular to compressive trabeculae and surrounded by BME-like signal [76] (see also “Stress response,” “Pathologic fracture”).

### Stress response

#### Pathogenesis

Stress response (stress reaction) is the failure of bone due to repetitive loading and cumulative microdamage. It represents an early stage of stress injury. Subclassifications include fatigue stress response (overuse activity in normal bone) and insufficiency stress response (normal activity in weakened bone) [31]. Whereas fatigue stress response tends to involve weight-bearing compact bone, insufficiency stress response tends to involve cancellous bone [76].

#### Imaging

Imaging findings may be localized to compact bone or cancellous bone. Radiographs are negative. Bone scintigraphy shows focal radiotracer uptake. On dual-energy CT, marrow fat may be effaced without visible cortical defect [38]. On MR, stress response and stress fracture fall along an imaging continuum and can be difficult to differentiate [74]. Stress injury is an appropriate term when imaging findings cannot distinguish stress response from fracture [98]. In stress response involving cortical bone, MR demonstrates endosteal and/or periosteal edema without cortical defect or fracture line [76]. In stress response involving cancellous bone, BME-like signal is similar in appearance to bone bruise (see also “Stress fracture,” “Bone bruise”).

### Subacromial shoulder impingement

See “Shoulder, subacromial impingement.”

### Subchondral insufficiency fracture

#### Histopathology

Subchondral insufficiency fracture (SIF) results from the mechanical failure of subchondral cancellous bone. Contributory factors include osteoporosis, obesity, overuse activity, and abnormal transarticular load distribution. Histologically, the fracture shows callus and nonmineralized osteoid without bone infarction [182]. Common locations include the femoral head, femoral condyle, and tibial plateau. SIF may be complicated by secondary osteonecrosis, osteonecrosis with cavitation (crescent sign), articular collapse and rapidly destructive arthropathy [183]. In the knee, secondary osteonecrosis may lead to the incorrect designation SONK (spontaneous osteonecrosis of knee) [183].

#### Imaging

Radiographs are often normal prior to healing callus formation or articular collapse. CT may show linear or patchy sclerosis involving subchondral cancellous bone. On MR, BME-like signal surrounds a fracture line. In contrast to the curvilinear boundary of osteonecrosis, insufficiency fracture parallels the subchondral bone plate without engaging it. And in contrast to an osteochondral defect, overlying cartilage remains intact. MR may show causative abnormalities (e.g., root avulsion of medial meniscus). When subchondral BME-like signal is present without definite fracture line, the differential diagnosis includes osteoarthritis, stress response, marrow contusion and, rarely, transient osteoporosis (see also “Crescent sign of osteonecrosis,” “Bone marrow edema,” “Transient osteoporosis”).

### Subluxation

#### Pathogenesis

Joint subluxation is the malalignment of articular surfaces. Whereas dislocation refers to complete (100%) loss of articular overlap, subluxation refers to partial loss. Subluxation is usually trauma-related, but it can be congenital (developmental dysplasia of the hip) or atraumatic (Ehlers–Danlos syndrome, neuromuscular imbalance). Traumatic subluxation is associated with ligamentous injury. Chronic subluxation suggests joint instability. Certain tendons may subluxate or dislocate (e.g., extensor carpi ulnaris, long head of biceps brachii, peroneus brevis and longus).

#### Imaging

Radiographs show articular incongruity. Subluxation may be mild (< 25% loss of overlap) or severe (> 75% loss of overlap). Provocative maneuvers and contralateral comparisons increase diagnostic sensitivity for subtle subluxation [184]. CT better demonstrates occult (Lisfranc fracture–subluxation) and complex (Chopart fracture–dislocation) fractures. In the elbow, shoulder, and knee, post-reduction radiographs may be negative despite extensive soft tissue injury [185]. MR shows patterns of bone marrow contusion and ligamentous injury that can clarify traumatic mechanism and help to differentiate transient subluxation from dislocation. Dynamic US enables stress maneuvers that provoke malalignment and prove joint instability.

### Swan neck deformity

#### Pathogenesis

Swan neck deformity is pathologic hyperextension of the PIP and flexion of the DIP joints of a finger. In the thumb, the metacarpophalangeal and interphangeal joints are affected. The alignment abnormalities are clinically obvious. Whereas boutonniere deformity is initiated by central slip tendon injury, swan neck deformity has multiple etiologies and potential mechanisms [186]. Either the PIP or DIP joint is affected primarily, followed by deformity of the other joint. The most common etiologies are rheumatologic (e.g., rheumatoid arthritis, systemic lupus erythematosus), neurologic (e.g., cerebral palsy, stroke, brain trauma), and posttraumatic (mallet finger, flexor digitorum superficialis tendon laceration, and PIP hyperextension) [43].

#### Imaging

Radiographs show PIP hyperextension and DIP flexion resembling a swan’s neck. Fractures (bony mallet, PIP volar plate avulsion) directly impact management and surgical treatment. MR demonstrates synovitis and tenosynovitis in rheumatoid arthritis. In traumatic tendon injury, MR delineates the extent of tear and degree of retraction (see also “Boutonniere deformity”).

### Synovitis

#### Histopathology

Synovitis is inflammation of the synovial-lined spaces of joints, bursae, or tendon sheaths. Acute and chronic inflammation occurs in both septic arthritis as well as immune-mediated arthropathies (e.g., rheumatoid arthritis, psoriatic arthritis, ankylosing spondylitis, reactive arthritis, and enteropathic arthritis). In chronic synovitis, hypervascular pannus is characterized histologically by villous hyperplasia, cellular infiltration, fibroblastic proliferation and, eventually, fibrosis. Synovitis is common in osteoarthritis and associated with both symptoms and disease progression.

#### Imaging

On noncontrast MR, inflammatory (exudative) and simple (transudative) effusions may be indistinguishable in the early stages of synovitis, bursitis, and tenosynovitis. On contrast MR, the inflamed synovial lining enhances [187]. This enhancement lacks diagnostic specificity and occurs in immune-mediated arthritis as well as septic arthritis, crystal deposition disorders, osteoarthritis, and joint trauma [43]. In chronic proliferative synovitis, pannus enhances on MR and demonstrates avid FDG uptake on PET due to increased cellular activity and glucose metabolism [188]. Pannus may erode cartilage and bone. When pannus shows decreased T2W signal, the differential diagnosis includes pigmented villonodular synovitis, hemophilic arthropathy, and amyloid. Gray-scale US and power Doppler can be used to monitor synovitis and treatment-related changes [189].

### Tendinopathy

#### Histopathology

Tendinopathy, or tendinosis, is a syndrome of localized tendon pain, tenderness, and swelling. Clinical diagnosis is based on functional criteria such as exercise intolerance. Predisposing factors include aging, overuse, and impingement. The relationships between symptoms, histopathology, and imaging are uncertain. Patients may be asymptomatic despite severe tendon degeneration. Histology initially shows tenocyte proliferation and adaptive chondrometaplasia. Connective tissue mucin, collagen fiber disruption, and variable capillary proliferation ultimately lead to frank mucoid change. Fatty deposits and tendinous calcifications may occur [73, 100, 190]. Altered mesenchymal cells express a variety of cytokines and matrix molecules responsible for inflammatory phenomena in the tendon and peritendinous tissues [73].

#### Imaging

Radiographs may show intratendinous mineralization (calcific tendinopathy) or ossification (ossific tendinopathy). US and MR depict tendon size, contour, and internal morphology. US demonstrates fibrillar disruption and hypoechoic heterogeneity. On color Doppler, hyperemia is commonly associated with tendinopathy of quadriceps, patellar, Achilles, and epicondylar tendons, but rarely the rotator cuff [191]. On contrast MR, hyperemic regions enhance [191]. Chronic insertional tendinopathy is associated with tendon tear, bony proliferation, and adjacent BME-like signal (see also “Paratenonitis,” “Enthesopathy ,” “Epicondylitis,” “Mucoid change”).

### Tendinosis

See “Tendinopathy.”

### Thigh splints

See “Femoral diaphyseal stress injury.”

### Tophus

#### Histopathology

In gout, monosodium urate crystals are deposited in cartilage, bone, and soft tissues. In tophaceous gout, a form of chronic gout, tophus refers to a mass-like core of monosodium urate crystals surrounded by a fibrovascular zone of acute and chronic granulomatous inflammation. Tophi usually involve joint capsules and juxta-articular tissues including tendons and ligaments. Common sites include first metatarsophalangeal joint, olecranon bursa, and extensor mechanism of knee. Tophi change in size with disease activity and treatment.

#### Imaging

Radiographs show eccentric, juxta-articular, mass-like nodules. Subjacent osseous erosions classically demonstrate sclerotic margins and overhanging edges. Cloud-like opacification reflects crystal deposition. CT is more sensitive for attenuation increases due to crystal deposition. Using dual-energy CT, decomposition algorithms color-code tophi for easy identification and volume quantification [192]. On US, tophi are heterogeneously hyperechoic with surrounding hypoechoic halo [193]. On color Doppler, this peripheral halo is often hypervascular. On MR, central low signal reflects the density of crystal deposition. Signal and contrast enhancement of the peripheral, fibrovascular zone depends on the degree of inflammation [194].

### Torsion

#### Pathogenesis

Torsion is a measure of rotation, or twisting, along the longitudinal axis of a bone. In long bones, torsional abnormalities are most common in the humerus, femur, and tibia and can result from developmental (positioning in utero), posttraumatic (fracture malunion), and overuse (repetitive throwing) etiologies [195]. Rotational osteotomy may be necessary to correct torsional deformities that would otherwise lead to joint derangements.

#### Imaging

On CT and MR, accurate, reproducible measurements of torsion angles require validated protocols, imaging planes, and anatomical landmarks [196]. The most important reference points are located at the ends of bones. The humerus normally shows retrotorsion between the humeral head and epicondyles. Retrotorsion is increased in throwing athletes [195]. The femoral neck normally demonstrates antetorsion relative to the condyles. Increased femoral antetorsion is associated with pincer-type femoroacetabular impingement (see also “Version,” “Glenoid retroversion,” “Acetabular retroversion”).

### Transient osteoporosis

#### Histopathology

Transient osteoporosis is an idiopathic, self-limited disorder affecting weight-bearing joints. The most common site is the femoral head. Histology shows nonspecific reparative bone remodeling. The main differential considerations are subchondral stress response and subchondral insufficiency fracture. Diagnostic difficulty results from overlapping clinical, imaging, and histopathological features [31, 197, 198]. Regional migratory osteoporosis is the term used to describe transient osteoporosis moving between joints.

#### Imaging

Radiographs show osteopenia 4–8 weeks after symptom onset. On bone scintigraphy, maximum uptake is seen in the femoral head, contrasting with the photopenic region seen in capital femoral necrosis. On MR, BME-like signal dissipates distally from subchondral bone without discrete fracture line or circumscribed osteonecrotic zone. Transient osteoporosis and subchondral stress response can share similar imaging appearances. Subchondral insufficiency fracture could be mistaken for transient osteoporosis if the fracture line is overlooked due to low-resolution imaging (see also “Stress response,” “Subchondral insufficiency fracture,” “Bone marrow edema”).

### Trochanteric syndrome

#### Pathogenesis

Trochanteric syndrome (greater trochanteric pain syndrome) is diagnosed clinically based on the presence of lateral hip pain overlying the greater trochanter. It commonly affects middle-aged females. Symptoms worsen with weight-bearing activity or lateral decubitus position. Etiologies include tendinopathy and insertional tears of gluteus minimus and/or medius. Gluteal tendon tears are associated with adjacent inflammation and greater trochanteric bursitis. Iliotibial band friction syndrome may cause identical symptoms.

#### Imaging

On radiographs, bony proliferative changes suggest gluteal ossific enthesopathy at greater trochanteric attachment sites. US and MR can depict tendinopathy and tears of gluteus minimus and medius [199]. Gluteal tendon tears are common in both symptomatic and asymptomatic patients [200]. Acute and chronic partial tears are associated with soft tissue inflammation and trochanteric bursal effusions. These findings are nonspecific and may also be seen in iliotibial band friction syndrome [91]. Acute osseous findings include BME-like signal. Chronic complete tears lead to gluteal fatty muscle atrophy (see also “Iliotibial band friction syndrome,” “Epicondylitis,” “Enthesopathy”).

### Tubulation

#### Anatomy

Tubulation refers to the size and shape of tubular bones. In children and adolescents, the modeling process regulates normal bone growth. Lengthening occurs at the epiphyseal plate (growth plate, physis). As a bone grows in length, the wide metaphysis must become tubular and transform into a narrow diaphysis. Therefore, osteoclastic resorption along the periosteal surface couples with osteoblastic bone formation along the endosteal surface. Final shaft diameter depends on appositional bone growth and the equilibrium between periosteal and endosteal bone resorption and formation. Bony remodeling continues after skeletal maturity.

#### Imaging

On radiographs, tubulation abnormalities are most evident in the long bones of the extremities [201]. Numerous factors, including mechanical forces, medications, and dysplasias (both hereditary and nonhereditary), can disrupt the balance in appositional bone resorption and formation. Overtubulation (gracile, narrowed form) is seen in osteogenesis imperfecta, neurofibromatosis, paralysis, and irradiation. Undertubulation (broad, widened form) is seen in Gaucher disease, thalassemia, and craniometaphyseal dysplasia [202].

### Tunnel syndrome

#### Pathogenesis

Tunnel syndrome refers to symptoms of pain, paresthesia, and weakness due to neurovascular compression, traction, or friction within a confined anatomical passageway. The passageway, or tunnel, may have a fibro-osseous or fibromuscular boundary. Carpal tunnel syndrome is the most common peripheral entrapment neuropathy. Mechanical entrapment also occurs within the tarsal tunnel, cubital tunnel, Guyon’s canal, and spinoglenoid notch. Electrodiagnostic testing (electromyography, nerve conduction study) and imaging studies support clinical assessment.

#### Imaging

On high-resolution US and MR, nerve swelling and hypervascularity correlate with neuropathic symptoms and abnormal electrodiagnostic testing [203, 204]. However, the nerve may have a normal appearance. MR neurographic parameters optimize contrast resolution and increase diagnostic sensitivity. Distal to the tunnel, muscle may show denervation edema. In chronic entrapment, fatty muscle infiltration is present. Space-occupying lesions within a tunnel include ganglion cyst, tenosynovitis, neoplasm, and accessory muscle or another structural variant.

### Ulnar impaction syndrome

#### Pathogenesis

Ulnar impaction syndrome (ulnocarpal impaction, ulnolunate abutment) refers to painful, excessive load across the ulna, triangular fibrocartilage, and ulnar carpus [205]. It is usually associated with positive ulnar variance. Acquired etiologies include distal radial fracture malunion, ulnar styloid fracture nonunion, radial head resection, and premature radial physeal closure. Chronic ulnocarpal abutment leads to triangular fibrocartilage tear and osteoarthritis. Ulnar impaction syndrome is the most common of the ulnar-sided impaction and impingement syndromes. It should be differentiated from the ulnar impingement syndrome which is caused by a shortened ulna that abuts and remodels the radius.

#### Imaging

On radiographs, supporting findings include positive ulnar variance and subchondral sclerosis at the ulnar side of lunate or radial side of triquetrum. To assess ulnar variance, the wrist must be properly positioned in neutral position with the shoulder abducted 90° and the elbow flexed 90°. Variance appears decreased in supination and increased in either pronation or fist clenching. In chronic impaction, radiographs may show ulnocarpal osteoarthritis [206]. Before the development of osseous changes on radiographs and CT, MR demonstrates degeneration or tearing of the triangular fibrocartilage and lunotriquetral ligament [206]. Associated MR findings include ulnocarpal chondromalacia, BME-like signal, and subchondral cyst-like change.

### Unhappy medial triad

See “O’Donoghue’s triad.”

### Version

#### Anatomy

Whereas torsion is a measure of the rotation of a long bone along its longitudinal axis, version is a measure of the rotation of a joint socket along its transverse axis. The combination of osseous torsion, version, and inclination affects joint stability and range of motion. Angulation (malrotation) abnormalities lead to mechanical dysfunction, instability, and impingement. Retroversion refers to abnormal posterior angulation of a joint socket, or cavity. It commonly affects the shoulder and hip. In the shoulder, glenoid retroversion predisposes to posterior glenohumeral instability and osteoarthritis. In the hip, acetabular retroversion predisposes to pincer-type femoroacetabular impingement.

#### Imaging

In the shoulder, glenoid version is measured on transverse CT or MR images. In the hip, acetabular version is assessed on radiographs and measured on transverse CT or MR images. CT 3D reconstructions may be useful for surgical planning (see also “Glenoid retroversion,” “Acetabular retroversion,” “Torsion”).

### Wolff’s law

#### Definition

Wolff’s law theorizes on the 3D architecture of bone [207]. Bone is a dynamic, metabolically active tissue that continuously reshapes itself. Bone structure adapts to physiological stress. Based on Wolff’s law, mechanical forces regulate the thickness of cortical bone as well as the thickness and geometric pattern of trabecular bone. Insufficient load (e.g., bedrest) leads to bone resorption and trabecular thinning. Excessive load (e.g., intense exercise) leads to bone formation along the lines of stress to distribute force and protect against stress fracture. Patterns of compressive and tensile trabeculae are most distinctive in the proximal femur and calcaneus.

#### Imaging

Bone quality and strength depend on both mass and structure. In clinical practice, bone mass is measured using bone mineral densitometry with either DXA or quantitative CT (QCT). In research and clinical trials, advanced techniques (e.g., high-resolution peripheral quantitative CT) can be applied to structural analysis and the assessment of trabecular architecture [129] (see also “Cancellous bone,” “Bone mineral density,” “Osteoporosis”).

## Abbreviations

BME: Bone marrow edema
CT: Computed tomography
MR: Magnetic resonance
T1W: T1-weighted
T2W: T2-weighted or fluid sensitive
US: Ultrasound
